# Neural correlates of overvaluation and the effort to save possessions in a novel decision task: An exploratory fMRI study

**DOI:** 10.3389/fpsyg.2023.1059051

**Published:** 2023-01-26

**Authors:** Tingting Liu, Brian D. Vickers, Rachael D. Seidler, Stephanie D. Preston

**Affiliations:** Department of Psychology, University of Michigan, Ann Arbor, MI, United States

**Keywords:** endowment, IKEA effect, decision making, hoarding disorder, rewards, emotion

## Abstract

**Introduction:**

People exhibit a strong attachment to possessions, observed in behavioral economics through loss aversion using new items in the Endowment or IKEA effects and in clinical psychology through pathological trouble discarding domestic items in Hoarding Disorder. These fields rarely intersect, but both document a reticence to relinquish a possessed item, even at a cost, which is associated with feelings of loss but can include enhanced positive states as well.

**Methods:**

To demonstrate the shared properties of these loss-related ownership effects, we developed the Pretzel Decorating Task (PDT), which concurrently measures overvaluation of one’s own over others’ items and feelings of loss associated with losing a possession, alongside enhanced positive appraisals of one’s items and an effort to save them. The PDT was piloted with 31 participants who decorated pretzels and responded to their own or others’ items during functional neuroimaging (fMRI). Participants observed one item per trial (self or other) and could work to save it (high or low probability loss) before learning the fate of the item (trashed or saved). Finally, participants rated items and completed hoarding tendency scales.

**Results:**

The hypotheses were supported, as even non-clinical participants overvalued, viewed as nicer, feared losing, and worked harder to save their items over others’—a response that correlated with hoarding tendencies and motor-motivational brain activation. Our region of interest in the nucleus accumbens (NAcc) was engaged when viewing one’s own items to the extent that people worked harder to save them and was more active when their items were saved when they felt emotionally attached to possessions in real life. When their items were trashed, NAcc activity negatively correlated with trouble discarding and emotional attachments to possessions. Right anterior insula was more active when working to save one’s own over others’ items. Extensive motor-motivational areas were engaged when working to save one’s own over others’ items, including cerebellum, primary motor and somatosensory regions, and retrosplenial/parahippocampal regions—even after controlling for tapping.

**Discussion:**

Our attachments to items are emotional, continuous across typical and pathological populations, and drive us to save possessions that we value.

## Introduction

Individuals spend significant time working for, buying, organizing, and discarding possessions. People vary widely, however, in the degree that they express this motivation to acquire and keep goods—from the most spartan among us, who keep few things and enjoy an uncluttered space to those suffering from pathological Hoarding Disorder (HD). Some have suggested that HD is an extreme form of people’s typical tendency to become attached to possessions and to want to hold on to them, even at a cost, but research linking these processes is limited and current findings are mixed. The primary goal of the present study was to directly test the relationship between typical endowment-like processes and continuous hoarding tendencies in non-patients using a novel fMRI task.

In behavioral economics, people often exhibit a strong or irrational attachment to possessions through the “endowment” or the “IKEA” effects. In the endowment effect, participants financially overvalue a new item like a mug that was just given to them by the experimenter, demonstrated when they require more money to sell the item than others are willing to pay for it (hereafter “overvaluation”) (Thaler, 1980; Kahneman et al., 1990, 1991). In the IKEA effect, people overvalue and appraise items to be nicer when they contributed to making them (Montgomery and Smith, 2008; Norton et al., 2012; Walasek et al., 2017). Both effects (hereafter “endowment processes”) are thought to reflect people’s ability to became quickly attached to items or feel some psychological ownership over them—an process that does not require literal ownership or physical possession (Morewedge et al., 2009). Research commonly suggests that endowment effects reflect our larger underlying decision bias called “loss aversion,” or the tendency to weigh losses more heavily than gains (Kahneman et al., 1991; Tversky and Kahneman, 1991; Ariely et al., 2005). Meanwhile, in clinical psychology, researchers have documented HD as the excessive acquisition, retention, and failure to discard even items of “useless” or “limited” value to others (Frost and Gross, 1993 p. 367; Preston et al., 2009; Preston and Vickers, 2014), which is distinct from Obsessive Compulsive Disorder (OCD) in the DSM-5 (Diagnostic and Statistical Manual of Mental Disorders, Fifth Edition; American Psychiatric Association, 2013).

There are clear similarities in the psychological and neural processes that underserve endowment and HD processes, which require further examination (reviewed in Preston and MacMillan-Ladd, 2021). Functionally, endowment and HD both involve a seemingly irrational tendency to hold on to a possession, often demonstrated when people are more reticent to discard a possession than to acquire a new one or discard an un-owned one. Studies of endowment and HD also both describe negative feelings like the fear of loss that promote this process more often than they describe the complementary positive states, such as positive appraisals of possessions and reward-based motivations to save them (but see Belk, 1988; Mellers and McGraw, 2001; Shu and Peck, 2011; Sokol-Hessner and Rutledge, 2019).

At its core, people can feel attached to a possession, similar to the way they can feel attached to a person, which inherently links the positive and rewarding feelings of love associated with a comforting target with inverse concerns about its loss (e.g., Ariely et al., 2005; Shu and Peck, 2011). Attachment as a concept was derived by John Bowlby and expanded by Ainsworth, Main, Shaver and others to classify attachment styles in infant-caregiver or romantic-partner dyads, which can predict responses to threat (e.g., Bowlby, 1969; Ainsworth, 1979; Main et al., 1985; Fraley and Shaver, 2000). Extensive research has similarly linked endowment and HD processes to people’s emotional attachments. For example, in typical populations, marketing research refers to “product attachment” as an emotional bond with a product that triggers emotion, feels special and significant, and leads to protection and care (Mugge et al., 2008). Russel Belk reviewed the many ways that people feel attached to possessions, which are part of their extended selves (Belk, 1988). Moreover, a set of nine studies demonstrated that the endowment effect *per se* is mediated through object attachment, operationalized as a sense of psychological ownership and an affective reaction to the item (Shu and Peck, 2011). In tasks where participants overvalue items that they helped make, researchers describe how “labor leads to love” (Norton et al., 2012). Extensive research also demonstrates people’s psychological ownership with goods, organizations, and the environment, which is associated with personal attachments, psychological closeness, high valuation, and anticipated loss—along with greater care and protection (Pierce et al., 2003; Baumeister and Wangenheim, 2014; Preston and Gelman, 2020). Psychological ownership is also exploited to render products more attractive (Baxter et al., 2015).

Attachment processes also support hoarding behavior. Hoarding tendencies increase the anthropomorphism of items, a love for them, a desire to be close to them, and a sense of responsibility for their care and protection (reviewed in Preston and MacMillan-Ladd, 2021). HD individuals report feeling soothed and comforted by their possessions, derive security from their fortresses of possessions, and grieve when items are removed (Frost and Steketee, 1999, 2010). The cognitive-behavioral model of HD includes intense emotional attachments to goods as a core belief, wherein emotional attachments are a stated reason to save goods, even endorsing items about loving “belongings the way I love some people” (Frost and Gross, 1993). The well-validated Savings and Cognition Inventory (SCI) predicts hoarding severity in patients and controls, including on the emotional attachment subscale (Steketee et al., 2003). Even in non-clinical individuals, hoarding tendencies increase emotional attachments to goods, comfort derived from them, and responsibility for their wellbeing (Frost et al., 1995; Phung et al., 2015). Directly linked to interpersonal attachment styles, possessions in HD are thought to compensate for poor interpersonal relationships (e.g., Frost and Hartl, 1996; Yap and Grisham, 2021). Higher hoarding tendencies are associated with more anxious and avoidant attachment styles, trouble regulating negative emotions, and greater emotional attachments to items (Medard and Kellett, 2014; Phung et al., 2015; Neave et al., 2016; Grisham et al., 2018; Norberg et al., 2018; Crone et al., 2019). Thus, extensive evidence supports similar processes that people use to maintain contact with goods as they do with bonded social partners, including a need for proximity that fosters security, comfort, love, and protection.

Some research has suggested that HD represents an extreme form of the more typical form of loss aversion or endowment. Clinical researchers have also suggested that HD is caused by the systematic overvaluation of items, which could augment typical endowment processes (Frost and Hartl, 1996; Tolin and Villavicencio, 2011; Wetzel, 2016; Pushkarskaya et al., 2020); however, results are mixed. For example, two clinical studies did not find a higher endowment effect in HD than healthy control participants, and endowment scores did not decline with successful treatment, even though they had moderate to large samples and tried two different tasks and (a traditional mug or chocolate task and a yard sale task). Perhaps indicating the more continuous nature of hoarding tendencies, scores across participants on a hoarding scale and its discarding subcale correlated with the endowment effect, particularly for items with more value (Pushkarskaya et al., 2020). Another study compared endowment in children with OCD (7 − 18yo) who had higher versus lower hoarding tendencies. This task initially endowed children with an item that they would subsequently keep or switch with another one of similar value (as in Knetsch, 1989; Harbaugh, 2001). Children with higher hoarding tendencies did more often keep their initial endowed item, but the analyses were non-parametric comparisons within groups that did not directly contrast groups, low hoarding children did not replicate the endowment effect, the sample was small, and there were no healthy control participants (Wetzel, 2016). OCD participants with hoarding symptoms were also more initially attached to a keychain endowed by the experimenters; this attachment remained stable over time (Grisham et al., 2009). When people with clinically-relevant hoarding tendencies were endowed with a human-like tea holder or box of tea, hoarding tendencies predicted attachment to items, which grew over a week, more so for people with anxious attachments (David et al., 2021).

It is reasonable to assume that HD represents the extreme end of a continuum that includes typical endowment processes lower down on the spectrum, since hoarding is an adaptive process that exists across species, exists across people on a spectrum, and exhibits similar psychopathological and behavioral patterns in control and HD participants (Frost et al., 1995; An et al., 2009; Preston et al., 2009; Norberg et al., 2015; Vickers et al., 2016). Further, many HD studies have successfully demonstrated hoarding-related issues using non-clinical participants (Preston et al., 2009; Tolin et al., 2009, 2012; Wang et al., 2012; Norberg et al., 2015; Shaw et al., 2015; Vickers et al., 2016). Because hoarding exists on a continuum, and this study includes non-clinical participants, hoarding symptom scores are referred to hereafter as “tendencies” so as not to imply that participant scores necessarily represent a problem.

Taken together, endowment and HD are similar processes, but research is needed that directly tests whether normal, individual differences in overvaluing possessions is associated with hoarding tendences. Moreover, we wanted to verify that this process involves both feelings of loss as well as reward-related appraisals and motivation. It is not possible to bridge the gap between endowment and HD processes using existing tasks. Endowment participants are nearly always offered money or must pay for an impersonal item that was just acquired (i.e., not a true “possession” as in HD, see also Ariely et al., 2005). Moreover, new items like mugs and pens could also be useful to other people (Kahneman et al., 1990). In contrast, HD studies that ask participants to discard real items from home are more ecological in the context of ownership but involve items without monetary value that are largely useless to others (e.g., junk mail or newspapers); as such, those tasks cannot compare the reticence to discard items with others’ desire to acquire them or with their monetary value. Thus, a task was needed that measures people’s reticence to give up or discard something they feel true ownership over, which also assesses monetary valuation of personal and of impersonal items that should also be useful to others.

To test our hypotheses, we developed a novel Pretzel Decorating Task (PDT) in which participants decorated a pretzel ahead of time and then, during functional neuroimaging (fMRI), viewed their own and others’ items, one at a time, could the work to save the item from the trash (high or low probability loss), and then learned the fate of the item (saved or trashed). People were expected to be willing to pay more for their items over others (as in endowment processes) and to rate the sense of loss as worse if they were to lose them, while also appraising them as nicer and being more willing to work to save them (associated with motor-motivational neural processes). This overvaluation was also expected to correlate with hoarding tendencies, even in our non-clinical sample using an HD symptom scale. We expected to replicate the link between expected loss and activation in the anterior insula observed in endowment effect (Knutson et al., 2008; Votinov et al., 2010) and HD discard tasks (Tolin et al., 2012). Item appraisal and successful saving were expected to activate the nucleus accumbens (NAcc), as also occurs in non-clinical endowment effect or shopping tasks (e.g., Knutson et al., 2007, 2008; Chib et al., 2009; De Martino et al., 2009; Hassall et al., 2016), in compulsive buying, which can co-occur with HD (Raab et al., 2011), and when people with higher hoarding tendencies acquire goods (Wang et al., 2012),

The PDT was expected to work because it resembled prior endowment and IKEA effect tasks that generally have large effect sizes. Moreover hoarding-related phenomena have been observed in non-patients previously and exist on a continuum. In addition, the use of a food item was expected to work because individual differences in hoarding tendencies previously correlated with intertemporal discounting for snack foods, even when identical items were offered to participants in large quantities (Vickers et al., 2016). Food also activates the decision and reward system that motivates organisms toward valued items (Chib et al., 2009)—regions that support decisions about goods and that correlate with hoarding tendencies (Preston, 2013a).

If we understand the underlying psychological, behavioral, emotional, and neural processes that cause people to overvalue possessions, we are in a better position to help people avoid maladaptive situations that can undermine financial security, well-being, and the health of the planet.

## Materials and methods

### Participants

Thirty-one participants (16 females) from the local community were recruited for a two-session study *via* flyers posted around campus and the community. The sample size was deemed adequate because fMRI results and relationships to hoarding had been established before using a similar task with a much smaller non-clinical sample (*N* = 20; Wang et al., 2012). We increased that sample by 50% to meet newer recommendations at the time for larger fMRI samples, but were still limited financially to 31 participants. This achieved 78% power using effect sizes from prior work on the endowment effect of 0.21 (Marzilli Ericson and Fuster, 2011; Camerer et al., 2016). Participants were compensated $10 for the pretzel-making session and $40 for the scanning session. All participants were 18–40 years of age (*M* = 20.97 years, *SD* = 4.29), right-handed, and without a history of neurological or psychiatric illness. All procedures were approved by the university Institutional Review Board, and written consent was obtained for each participant.

### The pretzel decorating task

A task that accesses both endowment and hoarding processes required real possessions that participants would value and want to save or fear losing, but still consent to lose. The items also had to be meaningful to owners and non-owners, and to be meaningful when presented in the multiples that were needed for fMRI. fMRI items also needed to be visually similar, to avoid visual confounds on brain activation. We also needed to measure feelings of loss along with positive appraisals and motivation toward items. An IKEA-like task suited these constraints (Norton et al., 2012). People should overvalue and become attached to items they helped decorate (compared to the one someone else decorated). Moreover, many similar looking snack items would be visually similar but people would still value them even in larger quantities (Haley and McKay, 2004). Thus, before scanning, participants decorated their own pretzels in the laboratory. On a subsequent day, they were tested at the fMRI center. During the task, they viewed one pretzel per trial (labeled as theirs or another’s) and could work to save the item from being trashed (with a high or low probability) and then learned the outcome (saved or trashed). After scanning, participants rated items across dimensions, performed a recognition task to demonstrate that they did recognize their items, and completed hoarding tendency scales.

### Procedure

**Figure 1:**
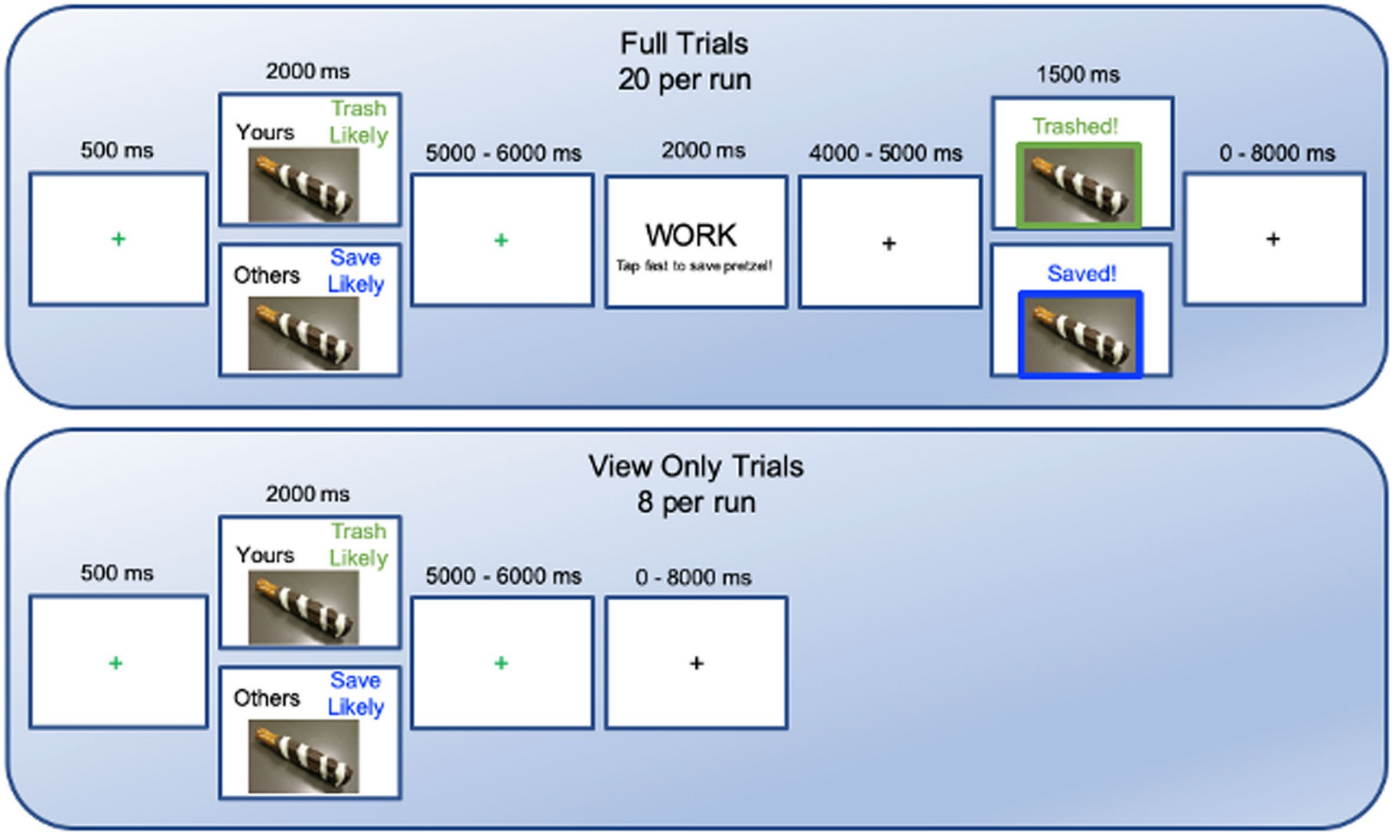
Sequence of events in the fMRI Pretzel decorating task.

One to two days before fMRI scans, participants consented and decorated 28 pretzel rods in the laboratory, by dipping them in melted white or milk chocolate and covering them with their preferred combination of crushed nuts, sprinkles, or miniature M&Ms. Pretzels were photographed individually with a Nikon Coolpix camera in a 4:3 size ratio, on the same gray surface, with the chocolate covered end facing the bottom right corner of the frame, going diagonally toward the top left, at approximately a 30° angle. Each participant’s items were slotted into their scanner trials, so personal items would appear in the same ratio for all participants. Others’ items were taken from a set created during a pilot pretzel-making session, to cover the range of quality observed by pilot participants. That is, pilot participants made a large set of pretzels and rated their quality from low to high, in terms of the subjectively perceived outcome of the decorating process. We then selected items for the other condition of low, medium, and high quality to match the range in future fMRI participants’ creations, based on the range in pilot participant creations. Using the same pilot items for the others’ pretzels across participants (rather than the actual other participants) was beneficial because we could institute the same delay between making the items and the fMRI visit for all participants (see Ariely et al., 2005; Grisham et al., 2009). The one-hour fMRI session included four runs of the PDT (Figure 1). There were also two tasks measuring brain activation during a simple “motor tapping” localizer and a “motor reaching imagery” localizer, to later subtract from our contrasts of interest (below and in the Supplementary material). After scanning, participants completed a computerized battery of pretzel rating and self-report questionnaires.

Task stimuli were presented using E-Prime 2.0 software projected onto a screen at the back of the magnet, viewed through a mirror. Pretzel stimuli varied on two orthogonal dimensions: creator (self-versus other) and loss probability (the likelihood that the pretzel would be thrown away at the end: high versus low). On each trial, subjects viewed one pretzel for 2000 ms, made by themselves or someone else, indicated with black text above and to the left of the image (“YOURS” or “OTHERS”). The top right displayed loss probability (“TRASH LIKELY” in olive text or “SAVE LIKELY” in blue). This was followed by a green jittered fixation cross on a white background for 5,000–6,000 ms.

For full trials, the next slide said “WORK” with “Tap fast to save pretzel!” underneath. Participants were instructed to tap faster during this 2000 ms period to the extent that they wanted to save the pretzel, to increase the likelihood that it would be saved. In reality, the number of trashed versus saved outcomes was predetermined (80% for the stated likelihood and 20% for the opposite) and participants could only take home their items because others’ items were the same for all participants. After tapping, a black jittered fixation cross appeared on a white screen for 4,000–5,000 ms to indicate the end of that period. Next the outcome slide presented that pretzel for 1,500 ms, surrounded by an olive border with the text “TRASHED” or a blue border with the text “SAVED!” above the image. Trials were separated by a black fixation cross on a white background that changed to green for the 500 ms before trial onset. No participants reported noticing that the outcomes were fixed.

The task intermixed view-only trials (~30%) and full trials that included work and outcome phases (~70%) to dissociate BOLD activity from viewing pretzels versus preparing to save them (after Ollinger et al., 2001a,b). View-only trials ended after the first green fixation cross (no work slide, second fixation, or outcome slide). Each of the four runs consisted of 28 trials (8 view-only; 20 full trials) split evenly among the four conditions (yours-save likely, yours-trash likely, other-save likely, other-trash likely). This produced 80 full trials for analysis. Each participant saw each of their 28 self-created pretzels twice, and the same 56 pretzels created by others once. Trial types were pseudorandomized per run with inter-trial intervals jittered between 0 and 8,000 ms (*M* = 1,640 ms, sampled from an exponential distribution). This allowed us to separately measure people’s response to observing their own or another’s item, aesthetic appeal, effects of loss probability, effort to save items, and the response to it being trashed or saved.

### Functional localizers

Participants completed a motor-tapping localizer after the Pretzel Decorating Task, to identify regions associated with simple tapping, to later subtract from activation during our Work period (i.e., above and beyond what is needed for the motor act). During three tapping blocks, participants saw a cursor blinking at 0.5 Hz, 2 Hz, or 4 Hz for 20 s and were asked to tap along with the cursor (randomized order). During rest periods between these blocks, participants relaxed for 20 s. The tapping localizer was followed by a motor reaching imagery localizer, in which participants imagined, in a 2 × 2 design, pulling a person or object back toward themselves, to rescue the item or for a more mundane reason. No brain areas were significantly more active for any contrast in this imagery localizer, which was included for a separate study on altruistic rescues and is not discussed further (described in the Supplementary material).

### Pretzel ratings

**Figure 2:**
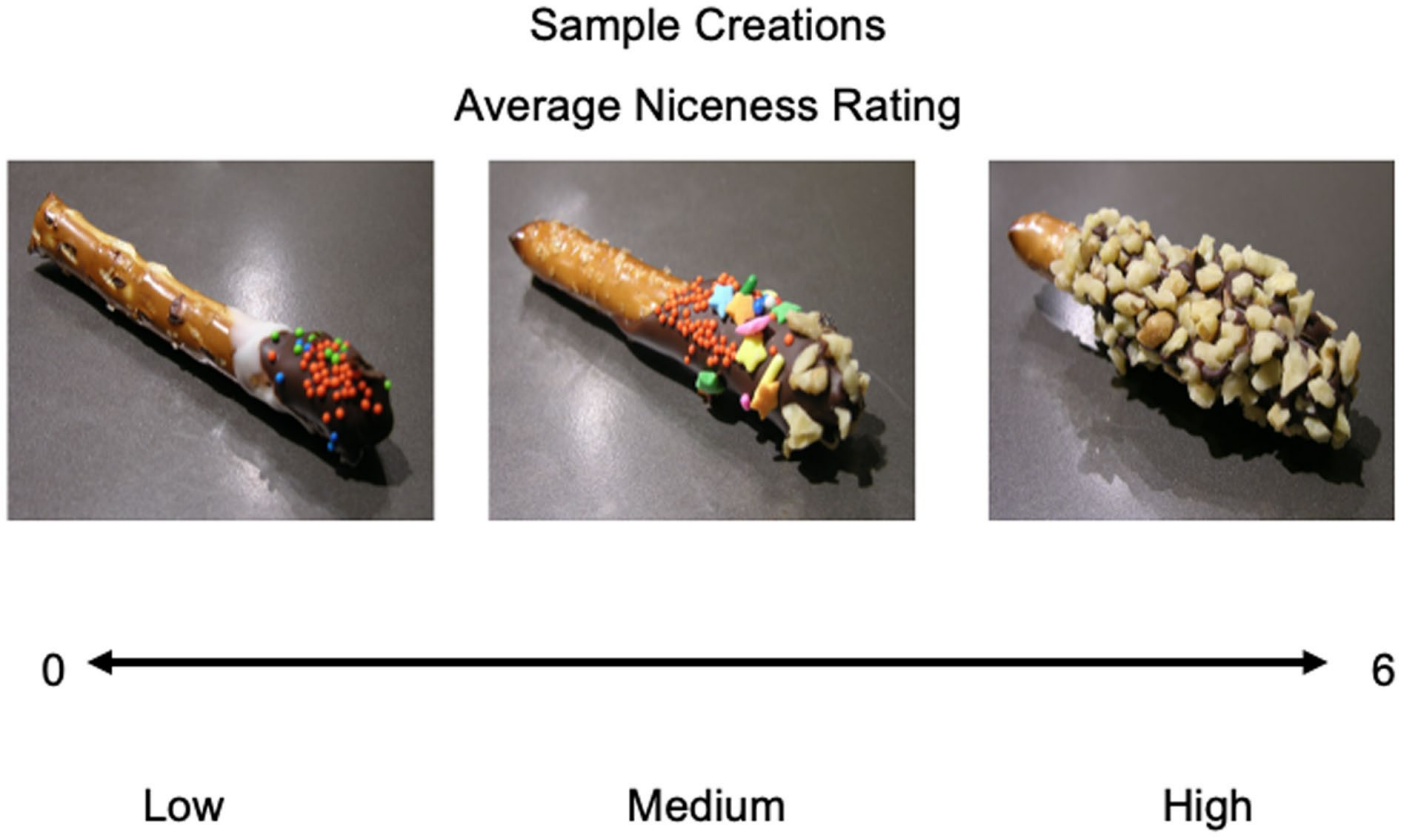
Sample pretzels rated on niceness. Participants rated their own and others’ pretzels that were shown in the scanner task from low (0) to high (6). Depicted are three sample items whose mean ratings across participants were low, medium, and high (left to right).

After scanning, participants rated each pretzel on (1) “Niceness”: how nice the pretzel looked (i.e., well made, attractive, professional looking; from 0 = Not nice at all to 6 = Very nice; Figure 2); (2) Willingness to pay (WTP): how much they would pay for the pretzel in cents; (3) Discard distress: how bad they would feel if the pretzel was thrown away or discarded (0 = Not bad at all to 6 = Very bad); and (4) The manipulation check: whether they thought the pretzel was their own, someone else’s, or unsure. Participants recognized their pretzels 75% of the time and ownership was labeled on every trial, ensuring that ownership was invoked.

### Individual-difference tendencies

Finally, participants completed a battery of questionnaires to relate performance on the task to individual differences in hoarding tendencies, including the Saving Inventory-Revised (SIR with subscales for over acquisition, trouble discarding, and clutter; Frost et al., 2004), Saving Cognitions Inventory (SCI with subscales for emotional attachment to items, memory, control, responsibility; Steketee et al., 2003), and Belk Materialism Scale (BMS with subscales for possessiveness, nongenerosity, envy; Belk, 1985). Behavior was correlated with all scales and subscales, but to reduce comparisons, relationships with brain activity were restricted to *a priori* hypotheses about the link between task variables and subscales that assessed retention issues: SIR-discard distress, SCI-emotional attachments, and BMS-possessiveness. Three additional scales were administered as pilot data for a separate study on altruistic rescues [see Supplementary material; Voluntary Simplicity Scale (VSC; Leonard, 1981); 30-item Penner Prosocial Battery (PSB; Penner et al., 1995); Locomotion Assessment Scale (LAS; Kruglanski et al., 2000)].

### Image acquisition

Imaging data were acquired using a 3.0 T GE Signa scanner with the standard head coil. To measure the blood oxygen level dependent (BOLD) signal for each participant in the main task, we acquired 1,060 functional T2* weighted spiral out volumes (slice thickness = 3 mm, 43 slices, repetition time [TR] = 2000 ms, echo time [TE] = 30 ms, flip angle [FA] = 90°, in plane resolution = 3.44 × 3.44 mm) divided evenly across four runs. Localizer tasks for motor tapping and motor reaching visualization were acquired with the same parameters but with 70 and 82 volumes, respectively. Trials were not initiated until after the first 5 functional images. Structural images for data presentation and co-registration were acquired in the same slice locations using a T1-weighted fast gradient echo pulse sequence (TE/FA = 30 ms/90 degrees, in plane resolution = 0.859 × 0.859 mm), and high-resolution structural images (voxel size 1 × 1 × 1 mm) were collected using a T1-weighted, spoiled 3D GRE acquisition.

### Behavioral analysis

Behavioral analysis consisted of six mixed models. We first compared participants’ ratings for each pretzel as the dependent measures in three separate linear mixed models: niceness, willingness to pay, discard distress. Participant was included as a random factor and pretzel creator as a fixed factor (self > other).

Next, in three similar linear mixed models we predicted participants’ behavioral tapping effect, that is, their greater effort to save one’s own over others’ pretzels in the 2 × 2 model with participant as a random effect. The number of taps were predicted in a linear mixed model with fixed factors for self > other and high > low loss probability (modeling each main effect and their interaction), including one covariate per model (pretzel niceness, willingness to pay, discard distress) and modeling the main effect of each rating and their interactions with self > other and high > low loss probability.

Hoarding tendencies were also modeled with a series of linear mixed models using the 2 × 2 model (self > other, high > low loss probability) with participant identity as a random effect, to predict the number of taps to save a pretzel as the dependent variable, with each hoarding tendency entered as a covariate. There was one model for each total score and for each subscale score, so the results were Bonferroni corrected for 21 comparisons (all scales and subscales). We report the main effect for each individual-difference tendency, their interaction with each main effect, and the three-way interaction (details in the Supplementary material).

### Brain imaging analysis

First-level analyses involve preprocessing data to correct for slice timing effects, motion, and to warp images to fit into a common Talairach template space in SPM12 (Statistical Parametric Mapping: The Analysis of Functional Brain Images - 1st Edition, 2007) using Matlab (MathWorks, Sherborn, MA). Images for analysis were smoothed at 4 mm Full Width at Half Maximum (FWHM) to compensate for inter-subject variability in the location of structures, reducing statistical noise in the activation. Statistical analysis was based on the general linear model, computing the BOLD signal contrast between creator (self, other), loss probability (high, low), and their interaction. Scans were resampled to 3 × 3 × 3 mm.

Second level analyses focused on key contrasts of interest in each of the three Pretzel Decorating Task periods: viewing one’s own versus others’ pretzels during Viewing, tapping to save valued pretzels during Work, and learning whether your items were saved or trashed during the Outcome (detailed below). Thresholding results of the 2 × 2 models first involved two-sided, one-sample *t*-tests in SPM with the default settings before estimating these models with the Threshold-Free Cluster Enhancement toolbox (TFCE^1^) with 5,000 permutations, using FWE correction, *k* > = 0. TFCE provides voxel-wise values that represent the amount of cluster-like local spatial support. The TFCE toolbox provides nonparametric estimation using TFCE for models estimated previously with SPM12 parametric statistics. TFCE was selected because it has been used widely and is more sensitive than other threshold methods without requiring arbitrary cluster formation thresholds (Smith and Nichols, 2009).

#### ROI analyses

For the *a priori* regions of interest (ROIs), we predicted NAcc and right anterior insula to be more active for self > other items, based on a neuroimaging study of the endowment effect (selling over buying in the whole brain; Knutson et al., 2008). To create the mask, NAcc peak coordinates were taken from this study, surrounded by a 20 mm sphere: NAcc left, (−11, 5, −3), NAcc right (8, 8, −2). The right anterior insula ROI was taken from voxels implied by the automated anatomical labelling atlas (AAL) for the right insula (Rolls et al., 2015) (Note: the NAcc was masked in both hemispheres but the insula only on the right, because only those three regions represented the endowment effect in Knutson et al., producing three clusters from two regions.)

Within the ROI, Viewing and Work periods included one key contrast that was also extracted to correlate with two additional variables. The first 2 × 2 model contrasted self > other creator crossed with high > low loss probability, thresholded at *p* < 0.1 FWE, k > = 0 in TFCE (based on three ROIs: R insula, R and L NAcc). Afterwards we extracted average brain activation within the ROI from the 2 × 2 model using MarsBaR (a toolbox for SPM providing routines for ROI analyses; (Brett et al., 2002) to correlate with the behavioral tapping effect (each person’s mean difference in tapping for self > other items) and with three hoarding tendency subscales that relate to difficulty parting with possessions (SIR-difficulty discarding, SCI-emotional attachment, and BMS-possessiveness). Correlations are reported with and without Bonferroni correction.

Analysis for the Work period included this 2 × 2 analysis and the correlations with the behavioral tapping effect and hoarding tendencies. Additionally, these results were masked with results from the simple tapping localizer. The tapping mask was derived from the 2 Hz motor tapping phase of the functional localizer (closest to the frequency of participants’ tapping during the Work period), thresholded at *p* < 0.001 uncorrected, k > = 10. These tapping-related areas were then excluded from self > other effects in the 2 × 2 model during Work, to yield regions that were additionally activated when trying to save items, above and beyond what is needed for the motor act of tapping, thresholded at *p* < 0.1 FWE TFCE, k > = 0.

The Outcome period permitted many possible contrasts, which we simplified by only performing those that reflected the psychology of losing or saving a possession in the ROI. We compared activation when one’s own items were discarded compared to all other possibilities (self trashed > all) and when one’s own items were saved versus trashed (self saved > self trashed) and correlated these effects with the three retention-related hoarding tendencies.

#### Whole brain analyses

Because this is a pilot study with only a moderate number of participants, it is possible that our *a priori* ROIs missed key areas of interest, producing multiple null results. Thus, ROI analyses were followed with exploratory whole-brain (WB) analyses, summarized below and detailed in the Supplementary material. WB analyses used the same 2 × 2 model from above, again thresholded at *p* < 0.1, FWE TFCE, k > = 0. Because people responded more to nicer looking pretzels in the behavioral results, we also explored null effects by adding a regressor for pretzel niceness to the 2 × 2 model in the WB. We report below the more stringent threshold of *p* < 0.05, FWE TFCE, k > = 0, but also include results in the supplement at the *p* < 0.10 level, FWE TFCE, k > = 0 (noting that this is still a more stringent threshold than uncorrected results). Null results were also explored by recalculating the 2 × 2 analyses in the whole brain for Viewing and Work with an alternative functional ROI from task > baseline (see the Supplementary material).

## Results

### Behavioral data

**Figure 3:**
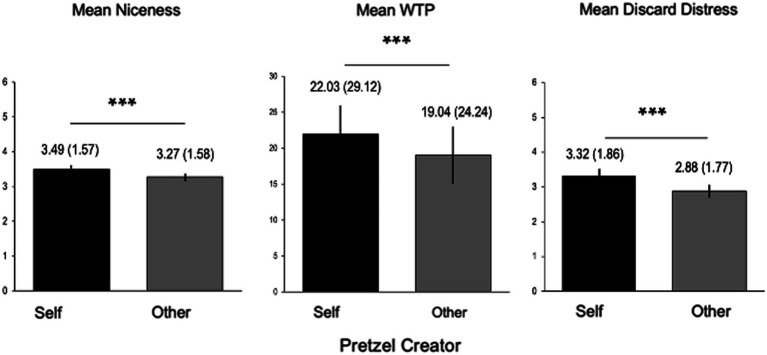
Self > Other rating overvaluation. Within-subject ratings on all dimensions increased for Self > Other. ***p < 0.001. WTP, Willingness to pay, in cents.

Supporting the key behavioral hypotheses, participants preferred their own items on every dimension to those made by others. Participants rated their items to be nicer than those created by others, *F*(1, 2,489) = 12.31, *p* < 0.001, they were willing to pay more for them, *F*(1, 2,489) = 22.78, *p* < 0.001, and they reported greater distress when imagining that they were discarded, *F*(1, 2,489) = 42.98, *p* < 0.001 (Figure 3).

**Figure 4:**
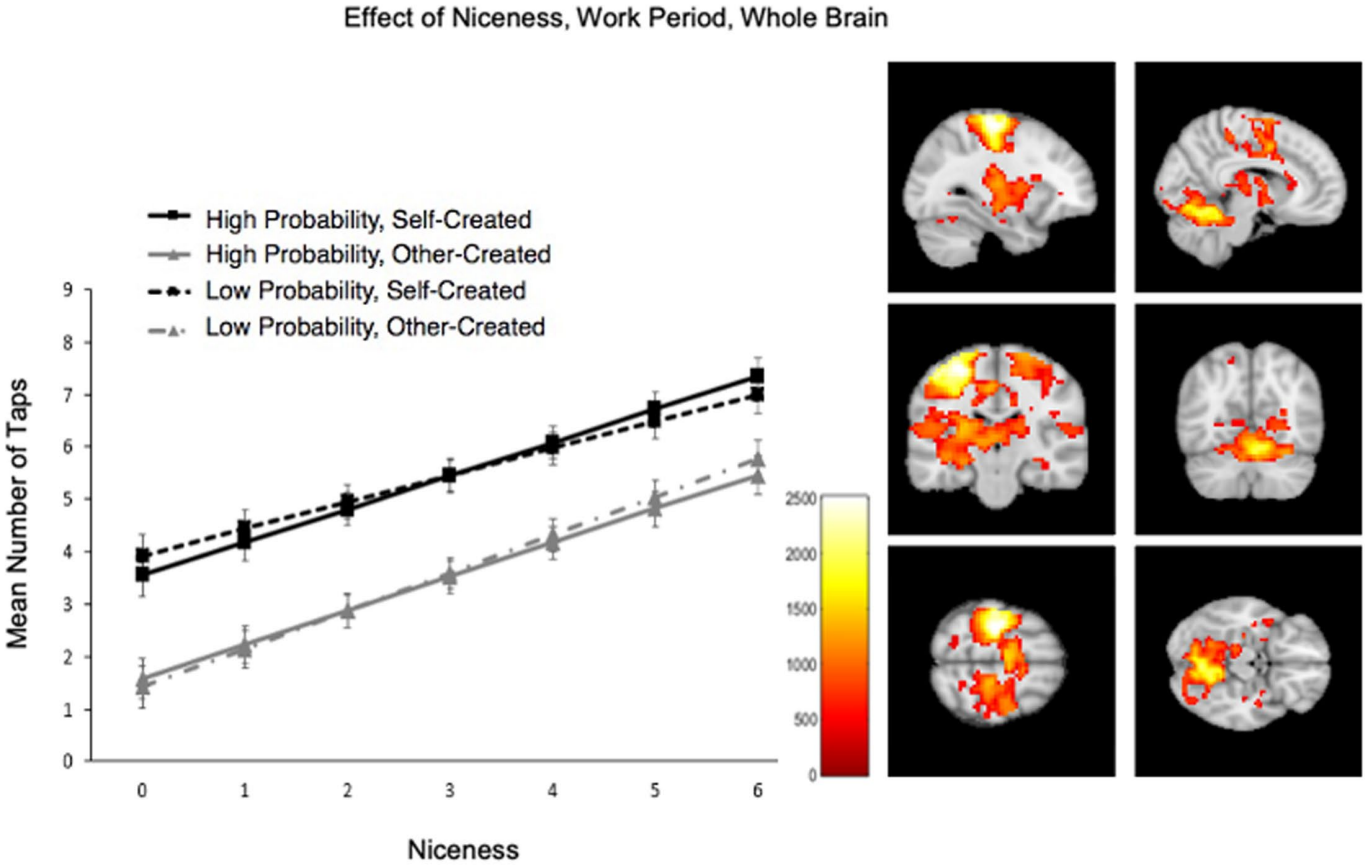
Increased effort for Self > Other and item niceness. Participants worked harder for their own over other’s items and for ones rated as nicer. Self items in black; Other in gray; dashed lines represent low loss probability; solid represent high loss probability. Brain activation from whole brain (WB) analysis during the Work period when tapping more for nicer pretzels.

Participants’ preference for self over other items was also reflected in how hard they worked to save them in the scanner (Figure 4). Participants worked harder for their own than others’ items, *M* (*SD*)*_self_* = 5.74 (0.31); *M* (*SD*)*_other_* = 3.72 (0.31), *F*(1, 2,322) = 297.59, *p* < 0.001. Across conditions, participants also worked harder for nicer pretzels, *F*(1, 2,339) = 274.741, *p* < 0.001, would pay more for them, *F*(1, 1930) = 115.90, *p* < 0.001, and reported being more distressed about imagining losing them, *F*(1, 2,342) = 317.19, *p* < 0.001. Note that this shows that participants not only prefer their pretzels because they decorated them to their own preferences, but that participants also agreed upon which were items were “nicer” and worked harder for all nicer ones, regardless of creator. Participants worked particularly hard for their pretzels to the degree that they would pay more for them, creator x WTP interaction: *F*(1, 2,324) = 10.08, *p* = 0.002. Effort to save was not influenced by whether the loss probability was high or low, *F*(1, 2,322) < 0.1, *p* > 0.1, and loss probability did not interact with any other effects across models, *F*s < 2.3, *p*s > 0.10.

As predicted, the behavioral tapping effect, to work harder to save one’s own over others’ items, also increased with multiple hoarding tendencies. The behavioral tapping effect was still significant after correcting for each hoarding tendency (except it did not survive correction after adding SIR-Acquisition), indicating that the impact of ownership does not just reflect the actions of unusual or disordered participants in our sample. Moreover, the behavioral tapping effect increased with continuous hoarding tendencies: SIR total scores, the SIR subscale for trouble with excess clutter in the home, and the SCI need for control over possessions (details in the Supplementary material).

### Brain imaging results

During simple viewing, no regions within the ROI were more active for self > other items (Table 1). However, left NAcc was more active during viewing one’s own items to the degree that participants subsequently tapped more for their items over others’ (Table 2). There was also a negative relationship between activation in right anterior insula and the hoarding tendency BMS-Possessiveness (Supplementary Table S3). No other comparisons were significant. Exploratory WB analysis found higher activation in left and right posterior cingulate cortex and precuneus when viewing self > other items (Table 2), with no additional impact of pretzel niceness (Table 2; Supplementary Table S4).

**Table 1:** ROI contrasts, per period, with the 2 × 2 model.

Period	Contrast	Region	Voxels	Peak equivZ	*x*	*y*	*z*
View	Self>Other		0				
Work	Self>Other	R AI	5	2.73	48	8	5
Outcome	Self trashed>All		0				
	Self saved>Self trashed	R NAcc	1	2.52	15	14	–1
			1	2.51	3	5	2

Threshold: *p* < 0.1 FWE TFCE, 0 voxels. R, Right; AI, Anterior Insula; NAcc, Nucleus Accumbens.

**Table 2:** Correlations of the behavioral tapping effect with 2 × 2 effects within the ROI, per period.

Period	Contrast	ROI	*r*	*p*
Viewing	Self > Other	L NAcc	0.45	0.012*^+^
		R NAcc	0.40	0.031*
		R AI	0.28	0.130
Work	Self > Other	L NAcc	0.11	0.583
		R NAcc	0.15	0.444
		R AI	0.42	0.020*
Outcome	Self trashed > All	L NAcc	−0.248	0.187
		R NAcc	−0.364	0.048*
		R AI	0.003	0.989
	Self saved > Self trashed	L NAcc	0.18	0.352
		R NAcc	0.23	0.217
		R AI	−0.07	0.731

L, Left; R, Right; AI, Anterior Insula; Nacc, Nucleus Accumbens. * = *p* < 0.05 before Bonferroni correction; ^+^ = still significant after correction.

During the work period, within the ROI, activation increased significantly for self > other items in a small cluster in right anterior insula (Table 1); this effect also increased with the behavioral tapping effect in right anterior insula, but the correlation did not survive correction, before correction *r* = 0.42, *p* = 0.02, after *p* = 0.06. Within the ROI, there were no other significant correlations between self > other activation and the behavioral tapping effect (Table 2) or hoarding tendencies (Supplementary Table S3).

Exploratory WB analysis during Work revealed significantly more activation for self > other during Work in broad regions associated with motor-motivational processes including cerebellum, left pre-and post-central gyrus (M1/S1), and right parahippocampal gyrus including retrosplenial cortex (Table 3). After masking out activation from simple motor execution from the motor tapping localizer, activation still increased significantly for self > other items in cerebellum and parahippocampal gyrus (Supplementary Figure S2; Supplementary Table S4). In the WB, during Work, activation for self > other items also increased with pretzel niceness in sensorimotor areas (left pre-and post-central gyri) and areas associated with visual memory (right parahippocampal gyrus, occipital gyrus, left posterior cingulate gyrus, and left precuneus; Table 3; Figure 4). Under a less conservative threshold, at *p* < 0.1 FWE TFCE, pretzel niceness additionally correlated during Work for self > other items in areas associated with object viewing and identification (e.g., superior and middle temporal gyri, middle frontal gyrus, and left middle and inferior occipital gyri; Supplementary Table S5).

**Table 3:** Whole-Brain contrasts using the 2 × 2 model, per period.

Period	Contrast	Region	Voxels	Peak equivZ	*x*	*y*	*z*
Viewing	Self > Other^1^	PCC/PCUN/RSC	35	2.3	−18	−55	23
			–	2.29	−12	−61	23
			–	2.58	−24	−61	20
			7	2.12	−9	−55	11
			2	2.49	27	−76	38
			1	2.65	18	−64	35
	Effect of niceness^2^		0				
Work	Self > Other^1^	Cerebellum	441	3.35	0	−61	−22
			–	3.09	9	−55	−16
			–	3.16	18	−49	−25
		M1/S1	256	2.71	−33	−28	56
			–	2.75	−30	−22	65
			–	2.73	−33	−16	56
		PHG/RSC	2	2.85	21	−46	11
			2	2.44	30	−55	8
			3	2.71	27	−49	5
	Effect of niceness^2^	M1/S1	9,215	3.54	−30	−22	59
			–	3.54	−27	−28	68
			–	3.54	−45	−13	53
		RSC	1	3.54	9	−40	−1
		PCC	3	3.16	−21	−34	38
		OCC	2	3.04	12	−97	5
		Caudate	1	3.35	−6	14	14
		PCUN/SPL	24	3.04	−12	−52	62
Outcome	Self trashed > All^1^		0				
	Self saved > Self trashed^1^		0				
	Effect of niceness^2^		0				

1 Threshold: *p* < 0.1 FWE TFCE, 0 voxels.

2 Threshold: *p* < 0.05 FWE TFCE, 0 voxels.L, Left; R, Right; M1, Primary Motor Cortex; S1, Primary Somatosensory Cortex; PCC, Posterior Cingulate Cortex; PCUN, Precuneus; PHG, Parahippocampal gyrus; OCC, Occipital Cortex/V1; RSC, Retrosplenial Cortex; SPL, Superior Parietal Lobule.

During the Outcome period, within the ROI, no regions were significantly more active when one’s own pretzels were trashed compared to all other possibilities, but there were significant negative correlations in left and right NAcc with hoarding tendencies for trouble discarding and emotional attachments to possessions (Supplementary Table S3; Supplementary Figure S3). When one’s own pretzels were saved versus trashed, activation increased in a small cluster in right NAcc (Table 1) and increased with emotional attachments to possessions in left NAcc (Supplementary Table S3). No other hoarding tendencies correlated with these contrasts within the ROI (Supplementary Table S3). WB analysis did not reveal any additional significant clusters, for either Outcome contrasts (Table 2) or correlations with pretzel niceness at either threshold (Table 2; Supplementary Table S4).

## Conclusion

People are often surrounded by things that they value and retain for a variety of reasons, beyond market value and simple utility, including personal attachments, associations with past memories, and representations of one’s identity (Belk, 1988; Frost et al., 1995; Steketee et al., 2003). Much of the existing research on our seemingly irrational attachment to our possessions comes from studies on the typical endowment or IKEA effects or pathological hoarding disorder (HD) (Vickers and Preston, 2014; Preston and MacMillan-Ladd, 2021). Both endowment and HD processes share a powerful resistance to letting go of a possessed item, even at a cost, which has been commonly associated with negative states like fear or “loss aversion” (Kahneman et al., 1991). Despite their similarities, efforts to link these processes have been limited, with mixed results.

We hypothesized that endowment and hoarding tendencies share an underlying mechanism, which includes the fear of losing a valued possession alongside a strong, positive appraisal of valued items that also motivates us to want to save them. To demonstrate a relationship between endowment and hoarding, while measuring both the negative and positive associations, we developed a novel Pretzel Decorating Task (PDT). This task combines the benefits of endowment and HD tasks because it uses items that people considered to be “theirs” and that they became attached, which are also be relevant to other people, even when offered in multiples. The task was supplemented with ratings to document people’s monetary valuation of their own and others’ items, negative feelings about potentially losing the items and their positive appraisals of them. Moreover, the task uniquely measures people’s behavioral effort to try to save their items from loss. Finally, all of these measures could be associated with non-clinical, individual differences in hoarding tendencies.

The task was also practical because it permitted multiple, repeated trials of saving and loss using visually similar items, as is required for fMRI. The fMRI design also allowed us to segregate activation associated with distinct phases of ownership including appraisal, effort to save the item, and the response to losing or saving it (Knutson et al., 2007). Because there was no monetary exchange, the task de-confounded monetary and possession processes that are combined in behavioral economics, which is important since money activates different mindsets than goods and has been proven to be less relevant to people with hoarding tendencies (Lea and Webley, 2006; Vickers et al., 2016). Statistically, the design also produces equal numbers of trials per condition and group, avoiding floor and ceiling effects that occur in HD studies, wherein controls discard most items and HD discard almost none.

This pilot study of the novel PDT was successful. Most importantly, it produced a strong endowment-like effect and clearly demonstrated people’s reticence to give up an item that they considered to be their own. Participants’ ratings also demonstrated, like in endowment tasks, that people monetarily overvalued their items over others’ and anticipated greater distress associated with their potential loss. In addition, we documented positive motor-motivational processes, as participants rated their items as nicer and physically worked harder in the scanner to try to save them from the trash. Finally, we were able to demonstrate the relationship between these forms of valuation and hoarding tendencies, across participant ratings, behavior, and in the brain. Taken together, the PDT has value as an ecologically-valid way to measure ownership that is amenable to testing in typical and pathological populations.

Our brain activation data were often null, in small regions, or in areas that we did not specify in our ROI. Generally, activation within our ROI (NAcc and insula) depended upon participant engagement and hoarding tendencies, in which case, effects may have been stronger with diagnosed HD patients. It is possible that participants were not motivated enough by the desert pretzels to engage these regions. For example, people vary in reward sensitivity and mesolimbocortical engagement increases to appetizing foods with participant individual differences in behavioral activation system (BAS) drive (i.e., the motivation to obtain what one wants; Beaver et al., 2006). This explanation does not seem to explain our results. We previously reported a link between impulsive choices to obtain food rewards and hoarding tendencies (Vickers et al., 2016) and tried to improve results by adding how nice each pretzel looked as a regressor. People did agree about which were nicer, they liked them more and they worked more for them; however, adding this variable did not improve ROI results. Moreover, Beaver and colleagues still observed significant group-level ventral striatum activation to appetizing food with half as many participants as we tested, despite BAS variability (but at a much lower striatal coordinate than ours). Whereas Beaver and colleagues contrasted appetizing with bland or disgusting foods, there was a desert pretzel on all of our trials, which could have led to a ceiling effect. Brain imaging results were much stronger during the work period, particularly for the exploratory whole-brain analyses, when participants tapped more to save their items, or nicer items, even after masking out brain activation that is required to tap at a similar rate. Thus, our task did still successfully measure the novel drive to save preferred items.

We did observe some support for the role of the NAcc, which was previously shown to mediate the endowment effect (Knutson et al., 2008; Votinov et al., 2010) and that we expected to support the positive, motivating rewards of possessions (Peters et al., 2003; Ariely et al., 2005; Shu and Peck, 2011; Preston and MacMillan-Ladd, 2021). Further research is needed. Our results were not uniform and often occurred in small clusters, potentially indicating a low-powered effect. The NAcc was not activated by the main contrasts of interest (e.g., self > other, save > trash) but it was sensitive to individual preferences and tendencies. For example, NAcc activation was higher during viewing one’s own items only after associating activity with one’s subsequent effort to save the item. In the outcome period, Nacc was only significantly more active after correlating responses with trait-like predispositions to want to hold onto possessions. Thus, not all items that are “ours” seem to operate the same way. Responses and corresponding brain activity depends on the personal value that we place upon possessions. The NAcc is part of a larger mesolimbocortical motor-motivational system that drives mammals toward desired items, from drugs of abuse to snack foods to attractive people to beautiful purses (Preston et al., 2014; Robinson et al., 2014). This region is also engaged when rodents hoard food and when they approach and retrieve helpless offspring; thus, this conserved mammalian circuit may promote actions to save both valued individuals and resources, through highly motivated processes (Preston, 2011, 2013a,b).

People did value nicer looking items more—both their own and others’—but efforts to save items remained higher for one’s own over others’ items across the spectrum of item quality, from least to most desired items. This demonstrates a role for positively appraising our own items during endowment and hoarding processes. Participants agreed upon which pretzels looked nicer and were willing to work harder for nicer ones whether they made them or not. This means that the overvaluation cannot just reflect the fact that one’s own items were catered to their idiosyncratic preferences (instead of ownership *per se*). Rather, preferences were largely similar across participants—demonstrating that the appraisals were not particularly idiosyncratic. Moreover, because effort was still always higher for one’s own items, the effect implicates ownership and the impacts of creation.

The right anterior insula was also part of our ROI because it was previously involved in both endowment tasks (Knutson et al., 2008; Votinov et al., 2010) and during discarding in HD (Tolin et al., 2012). As with the NAcc, anterior insula activity in our study was limited to smaller clusters that were not revealed until additional variables were added to ownership. During Viewing, the insula was more active for one’s own over others’ items when correlated with individual differences in possessiveness. During the Work period, activation increased in right anterior insula significantly for one’s own over others’ items, and this activation increased with the behavioral tapping effect (but the correlation with effort did not survive correction for multiple comparisons). Previously, HD studies found that, compared to participants with OCD and healthy controls, the insula was less active when people with HD were making decisions to discard others’ items but more active for their items; insula activation also increased with SIR hoarding severity scores and with “not just right” feelings when deciding to discard personal items (Tolin et al., 2012).

Supporting loss-aversion theories of ownership, research often describes the anterior insula as representing negative somatic states like pain, disappointment, or disgust (Wicker et al., 2003; Knutson et al., 2007, 2008; Votinov et al., 2010; Tong et al., 2016). The insula also informs decisions through somatic and affective inputs regarding choices and expected outcomes, for example, in risk prediction and risk prediction errors (Preuschoff et al., 2008), with monetary losses in a go-no-go task (Guitart-Masip et al., 2011), and tracking deviations from expected outcomes in the Ultimatum Game (Civai, 2013; Xiang et al., 2013). However, this region is also engaged for positive stimuli—with the exact location of activation changing along rostral-caudal and medial-lateral axes depending on stimulus valence and participant gender (Duerden et al., 2013). The insula likely serves a fundamental role in the awareness of interoceptive states more generally, engaged by both positive and negative experiences, across tasks, in ways that inform decisions (Craig, 2009). In our specific task the precise role of the insula is not yet clear, but like the NAcc, the insula appears to track individuals’ preferences and propensities more than the loss or reward associated with possession *writ large*.

Our exploratory whole-brain analyses revealed important regions that were activated by our task, which may track the effort to save items and the ability to remember creating them or to identify them individually. In our study, sensorimotor regions were activated when one could work to save their own over others’ items, even after controlling for motor tapping, and increased with item niceness. In prior HD studies, among HD participants, precentral gyrus and cerebellum activation was greater when participants refused to discard than successfully discarded their junk mail in the scanner (Tolin et al., 2009). In a similar task, the cerebellum was implicated when participants decided to discard their items compared to others’ items (Tolin et al., 2012).

In studies where participants imagine symptom-provocation scenarios in response to images, the precentral gyrus and cerebellum were significantly activated by the hoarding task in OCD hoarding participants, more so than in healthy controls; moreover, self-reported anxiety provoked by that task correlated with activation in pre-and post-central gyri and cerebellum across OCD patients (with and without hoarding) (An et al., 2009). Importantly, OCD hoarding individuals had lower cerebellar activation during the unrelated aversive control experiment than healthy control participants, indicating some context specificity to this response. A related study using the imagined symptom provocation task found more pre-central gyrus activation in patients with OCD during hoarding provocation (Mataix-Cols et al., 2004), and more pre-central and cerebellar activity in healthy controls doing the same task compared to neutral images across symptom types (washing, checking, hoarding) (Mataix-Cols et al., 2003). HD researchers have posited that this sensorimotor activity reflects the elevated anxiety or the requirement to press buttons, but our participants did not have an anxiety disorder and brain activation scaled with item niceness and remained after controlling for tapping. Thus, in the lab and the real world, a truly bodily motivation may be activated by the incentive to save a valued possession. Replications of the role of the cerebellum in discarding-related tasks in HD further support the need to upregulate motor-motivational processes when faced with the potential loss of an item and the continuity of processes between non-clinical and clinical populations. For example, the “effort-based decision-making” framework assumes that choices require actions that involve effort, the costs of which are integrated into preferences; people dis-prefer more effortful actions but select them when rewards are expected, mediated by dopaminergic motor-motivational processes (Kurniawan, 2011).

In addition to sensorimotor processes, regions that support visual processes, episodic memory, and object associations were involved in our task—which have been previously implicated in HD. In this study, a small region in parahippocampal gyrus and retrosplenial cortex was more active when participants worked for their items over others’ and for nicer items. In HD, this region was also more active in HD than healthy control participants when deciding whether to discard their junk mail or not (Tolin et al., 2009) and parahippocampal gyrus was also more active in HD than OCD patients when discarding personal and others’ items (Tolin et al., 2012). In the symptom provocation paradigm, the parahippocampal gyrus was more active in HD over non-HD participants, and increased with anxiety when imagining discarding goods (along with the amygdala/hippocampus complex) (An et al., 2009). This region was also more active when control participants viewed aversive compared to neutral images during symptom provocation of OCD-related symptoms (hoarding, checking, washing; Mataix-Cols et al., 2003). Activity in this region may, thus, reflect the fact that people are processing their episodic memory for the item and/or its identity, which becomes more salient when loss is possible (e.g., Vann et al., 2009).

There are some limitations to our study, which is natural given that this was a pilot demonstration of a task using a moderate number of participants. Researchers have suggested larger sample sizes for a robust test of individual differences (e.g., four times for an interaction of for a main effect; Gelman et al., 2020), for correlations, and for fMRI studies in general (Grady et al., 2021). Thus, our study should be replicated with a larger sample. This study should also be replicated in HD, to demonstrate that similar brain areas are involved because, for example, impacts of anxiety or the magical thinking in OCD-related hoarding may implicate different processes (Pertusa et al., 2008) and HD individuals may overvalue others’ items as well, which would obscure the key effect (e.g., Pushkarskaya et al., 2020).

We intentionally chose the framing of throwing the items into the trash, to try to maximize the effect size of the potential loss, but other frames should be used because oftentimes when people give up an item it can still retain some utility, which may be less distressing (e.g., when you sell the item, donate it to charity, or recycle it). We also intentionally used a food item because it easily allowed us to quickly attach people to items that would be considered theirs but that they would still consent to losing, and that could exist in replicates without diminishing returns (i.e., we only need so many mugs). We expect this response to generalize to material goods (e.g., Chib et al., 2009; Preston, 2011), but this should be demonstrated. We should also demonstrate the phenomenon with items taken from the home, to be continuous with work in HD. Our task could also be replicated with pre-made items assigned to participants as owners versus non-owners, to de-confound ownership and creation (i.e., the difference between the traditional endowment and IKEA effects).

In addition to the initial goal of using the Pretzel Decorating Task to study endowment effects and hoarding tendencies, the PDT is also well-suited to interrogate mesolimbocortical reward process more broadly, including in healthy control individuals and patients with a variety of conditions. For example, the PDT can be used to study disorders that involve anhedonia, trouble anticipating pleasure, low motivation, or trouble incorporating positive and negative feedback from impairment in the frontostriatal circuit, such as in depression, schizophrenia, Parkinson’s Disease (PD), substance abuse disorder, and subsequent to damage to the frontal lobe or striatum (e.g., Assogna et al., 2011; Dowd et al., 2016; Trøstheim et al., 2020; Shaw et al., 2021). For example, PD is characterized by dopaminergic denervation, leading to motor and cognitive impairment, which is treated with dopaminergic drugs like L-DOPA or DA agonists—treatment that can produce impulse control disorders (ICDs) including gambling, overeating, or hypersexuality (e.g., Weintraub, 2009; Djamshidian et al., 2013). As such, unmedicated patients can be hypo-motivated whereas DA-treated patients may be hyper-motivated (Ponsi et al., 2021). PD patients with ICDs are also more anxious and depressed and less able to learn from negative feedback (Martini et al., 2018) or to integrate changing sensory information toward advantageous decisions (Perugini et al., 2016). Similarly, with a modified urn task, PD patients with ICDs try to exit the task sooner after acquiring less information about marble color probability (Ruitenberg et al., 2022). During a set-shifting task, PD patients utilized feedback normally, but feedback use was inversely correlated with depression severity, particularly when the task was more difficult (Ravizza et al., 2012). This potential utility of our novel PMT to examine such impairments in PD is particularly relevant given the strong motor-cognitive activation during the work phase, which activated regions that are also implicated in PD, such as primary and secondary motor cortex (Kwak et al., 2012), fronto-parietal areas, cerebellum, bilateral striatum, middle frontal gyrus, and dorsal premotor cortex (Ruitenberg et al., 2022). The ecological nature of our task may also appreciated by patients and researchers alike given that aging, dementia, and other advanced stage-diseases are often characterized by under-stimulation and trouble understanding abstraction and response options used in traditional cognitive tasks (Cohen-Mansfield et al., 2010; Ravizza et al., 2012). The task may also be relevant for testing in PD and in disordered eating because of the specific use of food items. Finally, people with HD also often have comorbid depression and anxiety (Frost et al., 2011), which also co-occurs with PD in the presence of ICDs (Wu et al., 2015; Martini et al., 2018). Thus, it would be good to examine how disorders of emotion and motivation differentially affect phases of the task including appraisal, motivation, and response to feedback (see also Knutson et al., 2007). In sum, we designed the PDT to compare endowment effects and hoarding tendencies; however, the fact that the task is divided into consummatory phases makes it equally beneficial to study many other disorders.

This research should be extended to other socioeconomic groups or cultures, as this was a Western, American sample, taken from a population with relatively high wealth and education. It is likely that edible items that people create and their possessions more generally are valued in other cultures and contexts; however, more collectivistic or less materialistic cultures may not be as susceptible to effects of excessive acquisition, materialism, ownership, and personalization (see Markus and Kitayama, 1991).

There are a few effects that often occur in the lab or in the brain that we did not find. There were no effects of loss probability across our ratings or behavioral measures. One might suspect that this was not implicated because our actual outcomes were fixed, but this seems unlikely given that people did work harder for their items throughout the task, and it would be difficult to discern the disparity between reality and our largely accurate but fixed outcomes. This null effect might follow from the general tenant that loss aversion only holds for risks with an equal probability to win or lose (Novemsky and Kahneman, 2005), but future work should verify this. We also did not observe activation in the orbitofrontal cortex (OFC), which is commonly implicated during decision making in general, and even particularly in decisions about acquiring and discarding goods and hoarding tendencies (Anderson, 2004; Mataix-Cols et al., 2004; An et al., 2009; Tolin et al., 2009, 2012; Wang et al., 2012). Perhaps these null effects reflect the fact that this region is hard to scan because of its location or because we did not require the comparisons or trade-offs that are characteristic in decision-making tasks like the Iowa Gambling Task (Bechara, 2000) and when people can acquire or discard one of two items in a forced-choice task (Wang et al., 2012).

### Final comments

This study contributes to the literature by highlighting the degree that endowment and hoarding processes share attributes, involving both negative feelings of potential loss as well as positive, motor-motivational processes. These results cast ownership as more of a dynamic feedback loop, wherein the motivation to acquire something—and our attachment to it—fuel our effort to retain it and our reticence to let it go. This is a personal and relational process that is not purely “irrational” or “disordered,” and should not only be studied with money or in pathological cases. This is a normative process that is adaptive and that likely emerged from organisms’ important need to save resources and one another (Preston and Gelman, 2020; Preston and MacMillan-Ladd, 2021). These tendencies are largely adaptive in the big picture of evolution and human life, even if they sometimes cause trouble in a Western, industrialized context of overabundance.

## Data availability statement

The raw data supporting the conclusions of this article will be made available by the authors, without undue reservation.

## Ethics statement

The studies involving human participants were reviewed and approved by University of Michigan IRB. The patients/participants provided their written informed consent to participate in this study.

## Author contributions

TL was responsible for final data analysis and manuscript preparation. BV, RS, and SP contributed to study design and analysis plan. BV completed participant testing and initial data analysis and manuscript drafting. SP was responsible for funding, project supervision, and final manuscript preparation. All authors contributed to the article and approved the submitted version.

## Funding

This research was supported by a grant from the Templeton Foundation to SP and from the University of Michigan to SP; the funders were not involved in the study design, preparation, or submission.

## Conflict of interest

The authors declare that the research was conducted in the absence of any commercial or financial relationships that could be construed as a potential conflict of interest.

## Publisher’s note

All claims expressed in this article are solely those of the authors and do not necessarily represent those of their affiliated organizations, or those of the publisher, the editors and the reviewers. Any product that may be evaluated in this article, or claim that may be made by its manufacturer, is not guaranteed or endorsed by the publisher.

## Footnotes

^1^ http://dbm.neuro.uni-jena.de/tfce

## References

[ref1] Ainsworth M. S. (1979). Infant–mother attachment. Am. Psychol. 34, 932–937. doi: 10.1037/0003-066X.34.10.932 517843

[ref2] American Psychiatric Association, (2013). Diagnostic and Statistical Manual of Mental Disorders: DSM-5, Virginia: American Psychiatric Association.

[ref3] An S. K. Mataix-Cols D. Lawrence N. S. Wooderson S. Giampietro V. Speckens A.. (2009). To discard or not to discard: the neural basis of hoarding symptoms in obsessive-compulsive disorder. Mol. Psychiatry 14, 318–331. doi: 10.1038/sj.mp.4002129 18180763

[ref4] Anderson S. W. (2004). A neural basis for collecting behaviour in humans. Brain 128, 201–212. doi: 10.1093/brain/awh329 15548551

[ref5] Ariely D. Huber J. Wertenbroch K. (2005). When do losses loom larger than gains? J. Mark. Res. 42, 134–138. doi: 10.1509/jmkr.42.2.134.62283

[ref6] Assogna F. Cravello L. Caltagirone C. Spalletta G. (2011). Anhedonia in Parkinson’s disease: a systematic review of the literature. Mov. Disord. 26, 1825–1834. doi: 10.1002/mds.23815, PMID: 21661052

[ref7] Baumeister C. Wangenheim F. V. (2014). Access vs. ownership: understanding consumers consumption mode preference. SSRN Electron. J, 1–48. doi: 10.2139/ssrn.2463076

[ref8] Baxter W. L. Aurisicchio M. Childs P. R. N. (2015). A psychological ownership approach to designing object attachment. J. Eng. Des. 26, 140–156. doi: 10.1080/09544828.2015.1030371

[ref9] Beaver J. D. Lawrence A. D. van Ditzhuijzen J. Davis M. H. Woods A. Calder A. J. (2006). Individual differences in reward drive predict neural responses to images of food. J. Neurosci. 26, 5160–5166. doi: 10.1523/JNEUROSCI.0350-06.2006, PMID: 16687507 PMC6674259

[ref10] Bechara A. (2000). Emotion, decision making and the orbitofrontal cortex. Cereb. Cortex 10, 295–307. doi: 10.1093/cercor/10.3.295 10731224

[ref11] Belk R. W. (1985). Materialism: trait aspects of living in the material world. J. Consum. Res. 12, 265–280. doi: 10.1086/208515

[ref12] Belk R. W. (1988). Possessions and the extended self. J. Consum. Res. 15:139. doi: 10.1086/209154

[ref13] Bowlby J., (1969). Attachment and Loss, Attachment. New York: Basic Books.

[ref14] Brett M. Anton J.-L. Valabregue R. Poline J.-B., (2002). Region of interest analysis using an SPM toolbox, in 8th International Conference on Functional Mapping of the Human Brain. Sendai, Japan, p. 497.

[ref15] Camerer C. F. Dreber A. Forsell E. Ho T.-H. Huber J. Johannesson M.. (2016). Evaluating replicability of laboratory experiments in economics. Science 351, 1433–1436. doi: 10.1126/science.aaf0918 26940865

[ref16] Chib V. S. Rangel A. Shimojo S. O’Doherty J. P. (2009). Evidence for a common representation of decision values for dissimilar goods in human ventromedial prefrontal cortex. J. Neurosci. 29, 12315–12320. doi: 10.1523/JNEUROSCI.2575-09.2009, PMID: 19793990 PMC6666137

[ref17] Civai C. (2013). Rejecting unfairness: emotion-driven reaction or cognitive heuristic? Front. Hum. Neurosci. 7, 1–3. doi: 10.3389/fnhum.2013.00126, PMID: 23576973 PMC3617364

[ref18] Cohen-Mansfield J. Marx M. S. Dakheel-Ali M. Regier N. G. Thein K. (2010). Can persons with dementia be engaged with stimuli? Am. J. Geriatr. Psychiatry 18, 351–362. doi: 10.1097/JGP.0b013e3181c531fd, PMID: 20306565 PMC3142782

[ref19] Craig A. D. (2009). How do you feel — now? The anterior insula and human awareness. Nat. Rev. Neurosci. 10, 59–70. doi: 10.1038/nrn2555 19096369

[ref20] Crone C. Kwok C. Chau V. Norberg M. M. (2019). Applying attachment theory to indecisiveness in hoarding disorder. Psychiatry Res. 273, 318–324. doi: 10.1016/j.psychres.2019.01.055, PMID: 30677721

[ref21] David J. Aluh D. O. Blonner M. Norberg M. M. (2021). Excessive object attachment in hoarding disorder: examining the role of interpersonal functioning. Behav. Ther. 52, 1226–1236. doi: 10.1016/j.beth.2021.02.003, PMID: 34452675

[ref22] De Martino B. Kumaran D. Holt B. Dolan R. J. (2009). The neurobiology of reference-dependent value computation. J. Neurosci. 29, 3833–3842. doi: 10.1523/JNEUROSCI.4832-08.2009, PMID: 19321780 PMC2722101

[ref23] Djamshidian A. O’Sullivan S. S. Foltynie T. Aviles-Olmos I. Limousin P. Noyce A.. (2013). Dopamine agonists rather than deep brain stimulation cause reflection impulsivity in Parkinson’s disease. J. Parkinsons Dis. 3, 139–144. doi: 10.3233/JPD-130178, PMID: 23938343 PMC4205962

[ref24] Dowd E. C. Frank M. J. Collins A. Gold J. M. Barch D. M. (2016). Probabilistic reinforcement learning in patients with schizophrenia: relationships to anhedonia and avolition. Biol. Psychiatr. Cogn. Neurosci. Neuroimag. 1, 460–473. doi: 10.1016/j.bpsc.2016.05.005, PMID: 27833939 PMC5098503

[ref25] Duerden E. G. Arsalidou M. Lee M. Taylor M. J. (2013). Lateralization of affective processing in the insula. Neuro Image 78, 159–175. doi: 10.1016/j.neuroimage.2013.04.014 23587690

[ref26] Fraley R. C. Shaver P. R. (2000). Adult romantic attachment: theoretical developments, emerging controversies, and unanswered questions. Rev. Gen. Psychol. 4, 132–154. doi: 10.1037/1089-2680.4.2.132

[ref27] Frost R. O. Gross R. C. (1993). The hoarding of possessions. Behav. Res. Ther. 31, 367–381. doi: 10.1016/0005-7967(93)90094-B 8512538

[ref28] Frost R. O. Hartl T. L. (1996). A cognitive-behavioral model of compulsive hoarding. Behav. Res. Ther. 34, 341–350. doi: 10.1016/0005-7967(95)00071-2 8871366

[ref29] Frost R. O. Hartl T. L. Christian R. Williams N. (1995). The value of possessions in compulsive hoarding: patterns of use and attachment. Behav. Res. Ther. 33, 897–902. doi: 10.1016/0005-7967(95)00043-W, PMID: 7487849

[ref30] Frost R. O. Steketee G. (1999). Issues in the treatment of compulsive hoarding. Cogn. Behav. Pract. 6, 397–407. doi: 10.1016/S1077-7229(99)80058-3

[ref31] Frost R.O. Steketee G., (2010). Stuff: Compulsive hoarding and the meaning of things. Boston: Houghton Mifflin Harcourt.

[ref32] Frost R. O. Steketee G. Grisham J. (2004). Measurement of compulsive hoarding: saving inventory-revised. Behav. Res. Ther. 42, 1163–1182. doi: 10.1016/j.brat.2003.07.006, PMID: 15350856

[ref33] Frost R. O. Steketee G. Tolin D. F. (2011). Comorbidity in hoarding disorder. Depress. Anxiety 28, 876–884. doi: 10.1002/da.20861, PMID: 21770000 PMC3188689

[ref01] Gelman A. Hill J. Vehtari A. (2020). Regression and other stories. Cambridge University Press.

[ref02] Grady C. L. Rieck J. R. Nichol D. Rodrigue K. M. Tarkoff A. Kennedy K. M. (2021). Influence of sample size and analytic approach on stability and interpretation of brain‐behavior correlations in task‐related fMRI data. Hum. Brain Mapp. 42, 204–219. 32996635 10.1002/hbm.25217 PMC7721240

[ref34] Grisham J. R. Frost R. O. Steketee G. Kim H.-J. Tarkoff A. Hood S. (2009). Formation of attachment to possessions in compulsive hoarding. J. Anxiety Disord. 23, 357–361. doi: 10.1016/j.janxdis.2008.12.006, PMID: 19201154

[ref35] Grisham J. R. Martyn C. Kerin F. Baldwin P. A. Norberg M. M. (2018). Interpersonal functioning in hoarding disorder: An examination of attachment styles and emotion regulation in response to interpersonal stress. J. Obsess. Compul. Relat. Disord. 16, 43–49. doi: 10.1016/j.jocrd.2017.12.001

[ref36] Guitart-Masip M. Fuentemilla L. Bach D. R. Huys Q. J. M. Dayan P. Dolan R. J.. (2011). Action dominates valence in anticipatory representations in the human striatum and dopaminergic midbrain. J. Neurosci. 31, 7867–7875. doi: 10.1523/JNEUROSCI.6376-10.2011, PMID: 21613500 PMC3109549

[ref37] Haley L. McKay E. A. (2004). ‘Baking gives you confidence’: users’ views of engaging in the occupation of baking. Br. J. Occup. Ther. 67, 125–128. doi: 10.1177/030802260406700305

[ref38] Harbaugh W. T. (2001). Are adults better behaved than children? Age, experience, and the endowment effect. Econ. Lett. 70, 175–181. doi: 10.1016/S0165-1765(00)00359-1

[ref39] Hassall C. D. Silver A. Turk D. J. Krigolson O. E. (2016). We are more selfish than we think: the endowment effect and reward processing within the human medial-frontal cortex. Q. J. Exp. Psychol. 69, 1676–1686. doi: 10.1080/17470218.2015.1091849, PMID: 26490515

[ref40] Kahneman D. Knetsch J. L. Thaler R. H. (1990). Experimental tests of the endowment effect and the coase theorem. J. Polit. Econ. 98, 1325–1348. doi: 10.1086/261737

[ref41] Kahneman D. Knetsch J. L. Thaler R. H. (1991). Anomalies: the endowment effect, loss aversion, and status quo bias. J. Econ. Perspect. 5, 193–206. doi: 10.1257/jep.5.1.193

[ref42] Knetsch J. L. (1989). The endowment effect and evidence of nonreversible indifference curves. Am. Econ. Rev. 79, 1277–1284.

[ref43] Knutson B. Rick S. Wimmer G. E. Prelec D. Loewenstein G. (2007). Neural predictors of purchases. Neuron 53, 147–156. doi: 10.1016/j.neuron.2006.11.010, PMID: 17196537 PMC1876732

[ref44] Knutson B. Wimmer G. E. Rick S. Hollon N. G. Prelec D. Loewenstein G. (2008). Neural antecedents of the endowment effect. Neuron 58, 814–822. doi: 10.1016/j.neuron.2008.05.018, PMID: 18549791

[ref45] Kruglanski A. W. Thompson E. P. Higgins E. T. Atash M. N. Pierro A. Shah J. Y.. (2000). To “do the right thing” or to “just do it”: locomotion and assessment as distinct self-regulatory imperatives. J. Pers. Soc. Psychol. 79, 793–815. doi: 10.1037//0022-3514.79.5.793 11079242

[ref46] Kurniawan I. T. (2011). Dopamine and effort-based decision making. Front. Neurosci. 5, 1–10. doi: 10.3389/fnins.2011.00081, PMID: 21734862 PMC3122071

[ref47] Kwak Y. Peltier S. Bohnen N. Müller M. Dayalu P. Seidler R. D. (2012). L-DOPA changes spontaneous low-frequency BOLD signal oscillations in Parkinson’s disease: a resting state fMRI study. Front. Syst. Neurosci. 6, 1–15. doi: 10.3389/fnsys.2012.00052, PMID: 22783172 PMC3389385

[ref48] Lea S. E. G. Webley P. (2006). Money as tool, money as drug: the biological psychology of a strong incentive. Behav. Brain Sci. 29, 161–209. doi: 10.1017/S0140525X06009046, PMID: 16606498

[ref49] Leonard D. (1981). Voluntary simplicity lifestyles and energy consumption. J. Consum. Res. 8, 243–252. doi: 10.1086/208861

[ref50] Main M. Kaplan N. Cassidy J. (1985). Security in infancy, childhood, and adulthood: a move to the level of representation. Monogr. Soc. Res. Child Dev. 50:66. doi: 10.2307/3333827

[ref51] Markus H. R. Kitayama S. (1991). Culture and the self: implications for cognition, emotion, and motivation. Psychol. Rev. 98, 224–253. doi: 10.1037/0033-295X.98.2.224

[ref52] Martini A. Ellis S. J. Grange J. A. Tamburin S. Dal Lago D. Vianello G.. (2018). Risky decision-making and affective features of impulse control disorders in Parkinson’s disease. J. Neural Transm. 125, 131–143. doi: 10.1007/s00702-017-1807-7, PMID: 29119257 PMC5775350

[ref53] Marzilli Ericson K. M. Fuster A. (2011). Expectations as endowments: evidence on reference-dependent preferences from exchange and valuation experiments. Q. J. Econ. 126, 1879–1907. doi: 10.1093/qje/qjr034

[ref54] Mataix-Cols D. Cullen S. Lange K. Zelaya F. Andrew C. Amaro E.. (2003). Neural correlates of anxiety associated with obsessive-compulsive symptom dimensions in normal volunteers. Biol. Psychiatry 53, 482–493. doi: 10.1016/S0006-3223(02)01504-4, PMID: 12644353

[ref55] Mataix-Cols D. Wooderson S. Lawrence N. Brammer M. J. Speckens A. Phillips M. L. (2004). Distinct neural correlates of washing, checking, and hoarding symptom dimensions in obsessive-compulsive disorder. Arch. Gen. Psychiatry 61, 564–576. doi: 10.1001/archpsyc.61.6.564, PMID: 15184236

[ref56] Medard E. Kellett S. (2014). The role of adult attachment and social support in hoarding disorder. Behav. Cogn. Psychother. 42, 629–633. doi: 10.1017/S1352465813000659, PMID: 24103104

[ref57] Mellers B. A. McGraw A. P. (2001). Anticipated emotions as guides to choice. Curr. Dir. Psychol. Sci. 10, 210–214. doi: 10.1111/1467-8721.00151

[ref58] Montgomery A. Smith M. (2008). Prospects for personalization on the internet. J. Interact. Mark. 23, 130–137. doi: 10.2139/ssrn.1169874

[ref59] Morewedge C. K. Shu L. L. Gilbert D. T. Wilson T. D. (2009). Bad riddance or good rubbish? Ownership and not loss aversion causes the endowment effect. J. Exp. Soc. Psychol. 45, 947–951. doi: 10.1016/j.jesp.2009.05.014

[ref60] Mugge R. Schoormans J. P. L. Schifferstein H. N. J. (2008). “Product attachment: design strategies to stimulate the emotional bonding to products,” in Product experience. eds. P. Hekkert and H. N. J. Schifferstein (Elsevier),425–440.

[ref61] Neave N. Tyson H. McInnes L. Hamilton C. (2016). The role of attachment style and anthropomorphism in predicting hoarding behaviours in a non-clinical sample. Personal. Individ. Differ. 99, 33–37. doi: 10.1016/j.paid.2016.04.067

[ref62] Norberg M. M. Crone C. Kwok C. Grisham J. R. (2018). Anxious attachment and excessive acquisition: the mediating roles of anthropomorphism and distress intolerance. J. Behav. Addict. 7, 171–180. doi: 10.1556/2006.7.2018.08, PMID: 29444605 PMC6035017

[ref63] Norberg M. M. Keyan D. Grisham J. R. (2015). Mood influences the relationship between distress intolerance and discarding. J. Obsessive-Compuls. Relat. Disord. 6, 77–82. doi: 10.1016/j.jocrd.2015.06.005

[ref64] Norton M.I. Mochon D. Ariely D., (2012). The IKEA effect: When Labor Leads to Love, Boston: Harvard Business School.

[ref65] Novemsky N. Kahneman D. (2005). The boundaries of loss aversion. J. Mark. Res. 42, 119–128. doi: 10.1509/jmkr.42.2.119.62292

[ref66] Ollinger J. M. Corbetta M. Shulman G. L. (2001a). Separating processes within a trial in event-related functional MRI II. Anal. Neuro Image 13, 218–229. doi: 10.1006/nimg.2000.0711, PMID: 11133324

[ref67] Ollinger J. M. Shulman G. L. Corbetta M. (2001b). Separating processes within a trial in event-related functional MRI I. Method. Neuro Image 13, 210–217. doi: 10.1006/nimg.2000.0710 11133323

[ref68] Penner L. A. Fritzsche B. A. Craiger J. P. Freifeld T. R. (1995). Measuring the prosocial personality. Adv. Personal. Assess. 10, 147–163.

[ref69] Pertusa A. Fullana M. A. Singh S. Alonso P. Menchón J. M. Mataix-Cols D. (2008). Compulsive hoarding: OCD symptom, distinct clinical syndrome, or both? Am. J. Psychiatry 165, 1289–1298. doi: 10.1176/appi.ajp.2008.07111730, PMID: 18483134

[ref70] Perugini A. Ditterich J. Basso M. A. (2016). Patients with Parkinson’s disease show impaired use of priors in conditions of sensory uncertainty. Curr. Biol. 26, 1902–1910. doi: 10.1016/j.cub.2016.05.039, PMID: 27322000 PMC5633083

[ref71] Peters E. Slovic P. Gregory R. (2003). The role of affect in the WTA/WTP disparity. J. Behav. Decis. Mak. 16, 309–330. doi: 10.1002/bdm.448

[ref72] Phung P. J. Moulding R. Taylor J. K. Nedeljkovic M. (2015). Emotional regulation, attachment to possessions and hoarding symptoms. Scand. J. Psychol. 56, 573–581. doi: 10.1111/sjop.12239, PMID: 26183596

[ref73] Pierce J. L. Kostova T. Dirks K. T. (2003). The state of psychological ownership: integrating and extending a century of research. Rev. Gen. Psychol. 7, 84–107. doi: 10.1037/1089-2680.7.1.84

[ref74] Ponsi G. Scattolin M. Villa R. Aglioti S. M. (2021). Human moral decision-making through the lens of Parkinson’s disease. NPJ Park. Dis. 7:18. doi: 10.1038/s41531-021-00167-w, PMID: 33654110 PMC7925586

[ref75] Preston S. D. (2011). Toward an interdisciplinary science of consumption. Ann. N. Y. Acad. Sci. 1236, 1–16. doi: 10.1111/j.1749-6632.2011.06163.x 21883274

[ref76] Preston S. D. (2013a). “Hoarding in animals: the argument for a homology” in The Oxford Handbook of Hoarding and Acquiring. ed. Steketee R. O. F. G. (London: Oxford University Press)

[ref77] Preston S. D. (2013b). The origins of altruism in offspring care. Psychol. Bull. 139, 1305–1341. doi: 10.1037/a0031755, PMID: 23458432

[ref78] Preston S. D. Gelman S. A. (2020). This land is my land: psychological ownership increases willingness to protect the natural world more than legal ownership. J. Environ. Psychol. 70:101443. doi: 10.1016/j.jenvp.2020.101443

[ref79] Preston S. D. Kringelbach M. Knutson B. (2014). “Introduction” in The Interdisciplinary Science of Consumption. eds. Preston S. D. Kringelbach M. Knutson B. (Cambridge, MA: MIT Press)

[ref80] Preston S. D. MacMillan-Ladd A. D. (2021). Object attachment and decision-making. Curr. Opin. Psychol. 39, 31–37. doi: 10.1016/j.copsyc.2020.07.019, PMID: 32810749

[ref81] Preston S. D. Muroff J. R. Wengrovitz S. M. (2009). Investigating the mechanisms of hoarding from an experimental perspective. Depress. Anxiety 26, 425–437. doi: 10.1002/da.20417, PMID: 19242989

[ref82] Preston S. D. Vickers B. D. (2014). “The psychology of acquisitiveness” in The Interdisciplinary Science of Consumption. eds. Preston S. Kringelbach M. L. Knutson B. (Massachusetts, Cambridge: MIT Press)

[ref83] Preuschoff K. Quartz S. R. Bossaerts P. (2008). Human insula activation reflects risk prediction errors as well as risk. J. Neurosci. 28, 2745–2752. doi: 10.1523/JNEUROSCI.4286-07.2008, PMID: 18337404 PMC6670675

[ref84] Pushkarskaya H. Lenkic P. Stewart B. Tolin D. Woody S. R. (2020). Hoarding symptoms correlate with the endowment effect. J. Behav. Cogn. Ther. 30, 201–210. doi: 10.1016/j.jbct.2020.06.002

[ref85] Raab G. Elger C. E. Neuner M. Weber B. (2011). A neurological study of compulsive buying behaviour. J. Consum. Policy 34, 401–413. doi: 10.1007/s10603-011-9168-3

[ref86] Ravizza S. M. Goudreau J. Delgado M. R. Ruiz S. (2012). Executive function in Parkinson’s disease: contributions of the dorsal frontostriatal pathways to action and motivation. Cogn. Affect. Behav. Neurosci. 12, 193–206. doi: 10.3758/s13415-011-0066-6, PMID: 22006555

[ref87] Robinson M. J. F. Terry M. J. F. Berridge K. C. (2014). “Incentive salience in addiction and over-consumption” in The interdisciplinary science of consumption (Cambridge, MA: MIT Press)

[ref88] Rolls E. T. Joliot M. Tzourio-Mazoyer N. (2015). Implementation of a new parcellation of the orbitofrontal cortex in the automated anatomical labeling atlas. Neuro Image 122, 1–5. doi: 10.1016/j.neuroimage.2015.07.075, PMID: 26241684

[ref89] Ruitenberg M. F. L. Koppelmans V. Wu T. Averbeck B. B. Chou K. L. Seidler R. D. (2022). Neural correlates of risky decision making in Parkinson’s disease patients with impulse control disorders. Exp. Brain Res. 240, 2241–2253. doi: 10.1007/s00221-022-06423-6, PMID: 35852565 PMC10161684

[ref90] Shaw S. R. El-Omar H. Roquet D. Hodges J. R. Piguet O. Ahmed R. M.. (2021). Uncovering the prevalence and neural substrates of anhedonia in frontotemporal dementia. Brain 144, 1551–1564. doi: 10.1093/brain/awab032, PMID: 33843983

[ref91] Shaw A. M. Timpano K. R. Steketee G. Tolin D. F. Frost R. O. (2015). Hoarding and emotional reactivity: the link between negative emotional reactions and hoarding symptomatology. J. Psychiatr. Res. 63, 84–90. doi: 10.1016/j.jpsychires.2015.02.009, PMID: 25732668 PMC4387091

[ref92] Shu S. B. Peck J. (2011). Psychological ownership and affective reaction: emotional attachment process variables and the endowment effect. J. Consum. Psychol. 21, 439–452. doi: 10.1016/j.jcps.2011.01.002

[ref03] Smith S. M. Nichols T. E. (2009). Threshold-free cluster enhancement: addressing problems of smoothing, threshold dependence and localisation in cluster inference. Neuroimage 44, 83–98. 18501637 10.1016/j.neuroimage.2008.03.061

[ref93] Sokol-Hessner P. Rutledge R. B. (2019). The psychological and neural basis of loss aversion. Curr. Dir. Psychol. Sci. 28, 20–27. doi: 10.1177/0963721418806510

[ref94] Statistical Parametric Mapping: The Analysis of Functional Brain Images 1st edition [WWW document]. (2007). Available at: https://www.elsevier.com/books/statistical-parametric-mapping-the-analysis-of-functional-brain-images/penny/978-0-12-372560-8 (accessed 31 October, 2019).

[ref95] Steketee G. Frost R. O. Kyrios M. (2003). Cognitive aspects of compulsive hoarding. Cogn. Ther. Res. 27, 463–479. doi: 10.1023/A:1025428631552

[ref96] Thaler R. (1980). Toward a positive theory of consumer choice. J. Econ. Behav. Organ. 1, 39–60. doi: 10.1016/0167-2681(80)90051-7

[ref97] Tolin D. F. Kiehl K. A. Worhunsky P. Book G. A. Maltby N. (2009). An exploratory study of the neural mechanisms of decision making in compulsive hoarding. Psychol. Med. 39, 325–336. doi: 10.1017/S0033291708003371, PMID: 18485263

[ref04] Tolin D. F. Villavicencio A. (2011). An exploration of economic reasoning in hoarding disorder patients. Behav. Res. Ther. 49, 914–919. 21975192 10.1016/j.brat.2011.09.005 PMC3210419

[ref98] Tolin D. F. Stevens M. C. Villavicencio A. L. Norberg M. M. Calhoun V. D. Frost R. O.. (2012). Neural mechanisms of decision making in hoarding disorder. Arch. Gen. Psychiatry 69, 832–841. doi: 10.1001/archgenpsychiatry.2011.1980, PMID: 22868937 PMC3506167

[ref99] Tong L. C. P. Ye K. J. Asai K. Ertac S. List J. A. Nusbaum H. C.. (2016). Trading experience modulates anterior insula to reduce the endowment effect. Proc. Natl. Acad. Sci. U. S. A. 113, 9238–9243. doi: 10.1073/pnas.1519853113, PMID: 27482098 PMC4995964

[ref100] Trøstheim M. Eikemo M. Meir R. Hansen I. Paul E. Kroll S. L.. (2020). Assessment of anhedonia in adults with and without mental illness: a systematic review and meta-analysis. JAMA Netw. Open 3:e2013233. doi: 10.1001/jamanetworkopen.2020.13233, PMID: 32789515 PMC7116156

[ref101] Tversky A. Kahneman D. (1991). Loss aversion in riskless choice: a reference-dependent model. Q. J. Econ. 106, 1039–1061. doi: 10.2307/2937956

[ref102] Vann S. D. Aggleton J. P. Maguire E. A. (2009). What does the retrosplenial cortex do? Nat. Rev. Neurosci. 10, 792–802. doi: 10.1038/nrn2733 19812579

[ref103] Vickers B. D. Preston S. D. (2014). “The economics of hoarding,” in The Oxford handbook of hoarding and acquiring. eds. R. O. Frost and G. Steketee (Oxford University Press) 221–232.

[ref104] Vickers B. D. Preston S. D. Gonzalez R. Angott A. (2016). Hoarders only discount consumables and are more patient for money. Front. Hum. Neurosci. 10, 1–12.. doi: 10.3389/fnbeh.2016.00030 26973479 PMC4777727

[ref105] Votinov M. Mima T. Aso T. Abe M. Sawamoto N. Shinozaki J.. (2010). The neural correlates of endowment effect without economic transaction. Neurosci. Res. 68, 59–65. doi: 10.1016/j.neures.2010.05.006, PMID: 20538022

[ref106] Walasek L. Rakow T. Matthews W. J. (2017). When does construction enhance product value? Investigating the combined effects of object assembly and ownership on valuation. J. Behav. Decis. Mak. 30, 144–156. doi: 10.1002/bdm.1931

[ref107] Wang J. M. Seidler R. D. Hall J. L. Preston S. D. (2012). The neural bases of acquisitiveness: decisions to acquire and discard everyday goods differ across frames, items, and individuals. Neuropsychologia 50, 939–948. doi: 10.1016/j.neuropsychologia.2012.01.033, PMID: 22343029

[ref108] Weintraub D. (2009). Dopamine and impulse control disorders in Parkinson’s disease. Ann. Neurol. 64, S93–S100. doi: 10.1002/ana.21454, PMID: 19127573 PMC3530139

[ref109] Wetzel R. (2016). A higher endowment effect in children and adolescents with OCD and hoarding symptoms Dissertation/Thesis, McMaster University.

[ref110] Wicker B. Keysers C. Plailly J. Royet J.-P. Gallese V. Rizzolatti G. (2003). Both of us disgusted in my insula. Neuron 40, 655–664. doi: 10.1016/S0896-6273(03)00679-2, PMID: 14642287

[ref111] Wu K. Politis M. O’Sullivan S. S. Lawrence A. D. Warsi S. Bose S.. (2015). Single versus multiple impulse control disorders in Parkinson’s disease: an 11C-raclopride positron emission tomography study of reward cue-evoked striatal dopamine release. J. Neurol. 262, 1504–1514. doi: 10.1007/s00415-015-7722-7, PMID: 25893253

[ref112] Xiang T. Lohrenz T. Montague P. R. (2013). Computational substrates of norms and their violations during social exchange. J. Neurosci. 33, 1099–1108. doi: 10.1523/JNEUROSCI.1642-12.2013, PMID: 23325247 PMC3631781

[ref113] Yap K. Grisham J. R. (2021). Object attachment in hoarding disorder and its role in a compensatory process. Curr. Opin. Psychol. 39, 76–81. doi: 10.1016/j.copsyc.2020.07.022, PMID: 32853881

