# Supplementary material for: Neural correlates of overvaluation and the effort to save possessions in a novel decision task: An exploratory fMRI study
Source: Front Psychol. 2023 Jan 26;14:1059051. doi: 10.3389/fpsyg.2023.1059051 (PMC9911144; doi:10.3389/fpsyg.2023.1059051)
Supplement: Supplementary file 1 [file Table_1.docx]

**Supplement**

**Description of Motor Reaching Imagery Localizer**

In order to isolate areas of the brain involved in the active rescue of people, participants were asked to imagine four scenarios while in the scanner: two saving situations and two object situations. In the first saving situation, subjects were told to imagine that their friend had stepped into the road and hadn’t noticed an approaching car, and that they needed to reach out to pull their friend from danger. In the second saving situation, subjects were told to imagine they were fishing with a friend and that their friend had slipped into the river, and that they needed to reach down to pull their friend out.

In the first object situation, subjects were told to imagine reaching out and pulling a leaf off of a friend’s coat. In the second object situation, subjects were told to imagine they were fishing with a friend and needed to reach down to pick up a fishing pole off the ground. Participants were cued to visualize each situation with a short phrase. Each imagination block lasted for eight seconds, followed by eight seconds of rest. Each of the four blocks was repeated two times, giving us eight blocks for analysis. There were no significant results from this model, which was not central to our key questions, so it was not assessed further.

**Correlations with Individual Differences**

We administered a series of questionnaires that measure hoarding severity in psychopathology that can also be used as continuous measures or that correlate with hoarding tendencies in HD populations, to predict task and neural measures.

Saving Inventory Revised (SIR) [1]: a self-report measure validated for the assessment hoarding symptoms. It contains three subscales: Clutter, Acquisition, and Difficulty Discarding. The Saving Inventory Revised shows strong internal consistency, good reliability and validity in previous studies using both clinical and non-clinical samples (α′s ranging from 0.87 to 0.98; [1][7][8]).

Saving Cognitions Inventory Revised (SCI) [2]: a measure of attitudes and beliefs among compulsive hoarders. This inventory has good convergent and discriminant validity, and internal consistency in both clinical and nonclinical samples (*α* ranging from 0.86 to 0.96; [2][9])

Belk Materialism Scale (BMS) [3]: a widely used 5-point Likert scale measuring individual differences in materialism (possessiveness, nongenerosity, envy), including 24 items. This scale has been widely used in the literature to measure the materialism and has acceptable retest reliability (*α* above 0.6; [2])

Voluntary Simplicity Scale (VSS) [4]: an 18-item scale was used to measure participants’ engagement in simplifying behaviors related to proenvironmentalism. Participants rate the behavioral frequency for each item, for example, “Recycle newspapers used at home” or “Ride a bicycle for transportation to work”. It has good validity [4][10]. We did not analyze data from this scale to simplify analysis; it was intended as a pilot measure for other studies on the overlap between people’s motive to save non-human, human, and environmental targets, which was irrelevant with a nonsignificant motor reaching imagery localizer.

Table S1 includes results from adding each hoarding tendency to the main 2 x 2 model to predict the behavioral tapping effect (self > other by high > low loss probability by hoarding tendency). Each linear mixed model added one total or subscale score and re-produced the behavioral outcomes for self > other, high > low loss probability and their interaction, alongside a result for each hoarding tendency for the main effect of the hoarding tendency, the interaction with self > other items and with high > low loss probability, as well as the three-way interaction. Results were Bonferroni corrected for 21 scale totals and subscales, including the prosocial tendency scores described below. The key self > other impact on tapping to save items was still significant after entering all of the hoarding tendencies (except that SIR-Acquisition did not survive Bonferroni correction). Multiple hoarding tendencies also interacted with this effort to save one’s own items over others’; two survived Bonferroni correction: SIR total scores, SIR excess clutter subscale, and SCI need for control over items.

**Table S1**

**Impact of each hoarding tendency on the 2 x 2 model to predict the behavioral tapping effect**

| **Contrast** | **Moderator** | ***F*** | ***p*** | **Sig** |
| --- | --- | --- | --- | --- |
| Self > Other X Hoarding Tendency | BMS-Envy | 1.18 | 0.28 |  |
|  | BMS-Non-generosity | 0.03 | 0.87 |  |
|  | BMS-Possessiveness | 0.26 | 0.61 |  |
|  | BMS-Total | 0.82 | 0.37 |  |
|  | **SCI-Control** | **10.26** | **0.00** | ****** |
|  | SCI-Emotional Attachment | 2.75 | 0.10 |  |
|  | SCI-Memory | 1.96 | 0.16 |  |
|  | SCI-Responsibility | 0.60 | 0.44 |  |
|  | SCI-Total | 2.87 | 0.09 |  |
|  | SIR-Acquisition | 5.43 | 0.02 | * |
|  | **SIR-Clutter** | **9.53** | **0.00** | ****** |
|  | SIR-Discard | 7.24 | 0.01 | ** |
|  | **SIR-Total** | **11.47** | **0.00** | ******* |
| High > Low Loss Probability X Hoarding Tendency | BMS-Envy | 2.65 | 0.10 |  |
|  | BMS-Non-generosity | 2.07 | 0.15 |  |
|  | BMS-Possessiveness | 0.02 | 0.89 |  |
|  | BMS-Total | 2.07 | 0.15 |  |
|  | SCI-Control | 0.41 | 0.52 |  |
|  | SCI-Emotional Attachment | 1.75 | 0.19 |  |
|  | SCI-Memory | 0.34 | 0.56 |  |
|  | SCI-Responsibility | 0.00 | 1.00 |  |
|  | SCI-Total | 0.52 | 0.47 |  |
|  | SIR-Acquisition | 5.64 | 0.02 | * |
|  | SIR-Clutter | 1.52 | 0.22 |  |
|  | SIR-Discard | 0.57 | 0.45 |  |
|  | SIR-Total | 2.77 | 0.10 |  |
| Self > Other X High > Low Loss Probability X Hoarding Tendency | BMS-Envy | 0.91 | 0.34 |  |
|  | BMS-Non-generosity | 2.94 | 0.09 |  |
|  | BMS-Possessiveness | 6.53 | 0.01 | * |
|  | BMS-Total | 2.11 | 0.15 |  |
|  | SCI-Control | 4.87 | 0.03 | * |
|  | SCI-Emotional Attachment | 3.49 | 0.06 |  |
|  | SCI-Memory | 3.90 | 0.05 | * |
|  | SCI-Responsibility | 2.66 | 0.10 |  |
|  | SCI-Total | 4.45 | 0.03 | * |
|  | SIR-Acquisition | 1.63 | 0.20 |  |
|  | SIR-Clutter | 3.67 | 0.06 |  |
|  | SIR-Discard | 0.10 | 0.76 |  |
|  | SIR-Total | 2.40 | 0.12 |  |

*Note.* Correlations between Creator and SCI-Control, SIR-Clutter, and SIR-Total survived Bonferroni correction (bolded).

***: *p* < 0.001, **: *p* < 0.01, *: *p* < 0.05

BMS-Envy: Belk Materialism Scale-Envy; BMS-Non-generosity: Belk Materialism Scale-Non-generosity; BMS-Possessiveness: Belk Materialism Scale-Possessiveness; BMS-Total: Belk Materialism Scale-Total; SCI-Control: Saving Cognitions Inventory-Control; SCI-Emotional Attachment: Saving Cognitions Inventory-Emotional Attachment; SCI-Memory: Saving Cognitions Inventory-Memory; SCI-Responsibility: Saving Cognitions Inventory-Responsibility;

SCI-Total: Saving Cognitions Inventory-Total; SIR-Acquisition: Saving Inventory Revised-Acquisition; SIR-Clutter: Saving Inventory Revised-Clutter; SIR-Discard: Saving Inventory Revised-Discard; SIR-Total: Acquisition Saving Inventory Revised-Total.

We also administered two additional trait scales in case the act of wanting to “save” one’s own pretzel was similar to that of saving a person: the 30-item Penner Prosocial Battery ([PSB);](#_heading=h.3dy6vkm) Penner LA, Fritzsche BA, Craiger JP, Freifeld TR., 1995) and the Locomotion Assessment Scale ([LAS; Kruglanski et al., 2000](#_heading=h.1t3h5sf)). Because the saving localizer did not produce significant results, these scales also were not considered central to our analyses.

Penner Prosocial Personality Battery (PSB) [5]: a 30-item scale that assess the personality variables in helping. It provides evidence that a prosocial personality may in fact lead people to seek out volunteer opportunities. It consists of six subscales: empathic concern, helpfulness, personal distress, perspective taking, social responsibility, self-reported altruism. It has been shown to have a good internal consistency (Cronbach’s alphas > 0.7 [11])

Locomotion Assessment Scale (LAS) [6]: a 6-point Likert scale used to assess individual differences in self-regulation. It includes two 12-item subscales for locomotion (e.g., When I get started on something I usually persevere until I finish it”) and assessment (e.g.,

I am a critical person”). Both subscales show satisfactory reliability and internal consistency (Cronbach’s alphas > 0.7 [6][12]).

Using the same model as for the hoarding tendencies (scales correlated with added effort for self > others’ items in the 2 x 2 model for self or other; high or low loss), the more people reported perspective taking in daily life, they less they worked to save their pretzels and more to save another’s, PSB- perspective taking: *F* (1, 2319) = 15.86, *p* < 0.001. A significant association with LAS-Locomotion showed that higher tendencies produced less effort for their own and other’s pretzels, but with a greater decrease for their own (Table S2).

Two subscales significantly moderated the interaction between self > other and high > low loss probability after Bonferroni correction: PSB-Empathic Concern and LAS-Locomotion,. The more empathic concern people reported in daily life, the less they worked to save their own pretzels if it was likely to be trashed. For others’ pretzels, they worked a similar amount over regardless of trait locomotion if it was likely to be trashed but worked less for others’ pretzels that were likely to be saved. If the pretzels were likely to be trashed, people with greater locomotion tendencies worked less for their own pretzels, but about the same for others’. If the pretzels were likely to be saved, they worked less for both their own and others’ pretzels.

**Table S2**

**Impact of each prosocial tendency on the 2 x 2 model to predict the behavioral tapping effect.**

| **Effect Moderated** | **Moderator** | ***F*** | ***p*** | **Sig** | **Survived**  **Bonferroni** |
| --- | --- | --- | --- | --- | --- |
| Self > Other X Prosocial Tendency | LAS-Assessment | 0.62 | 0.43 |  |  |
|  | **LAS-Locomotion** | **12.54** | **0.00** | ******* | **Y** |
|  | PSB-EC | 1.72 | 0.19 |  |  |
|  | PSB-Helpfulness | 1.06 | 0.30 |  |  |
|  | PSB-PD | 2.85 | 0.09 |  |  |
|  | **PSB-PT** | **15.86** | **0.00** | ******* | **Y** |
|  | PSB-SR | 0.89 | 0.35 |  |  |
|  | PSB-SA | 4.96 | 0.03 | * |  |
| High > Low Loss Probability X Prosocial Tendency | LAS-Assessment | 0.36 | 0.55 |  |  |
|  | LAS-Locomotion | 1.10 | 0.29 |  |  |
|  | PSB-EC | 0.03 | 0.86 |  |  |
|  | PSB-Helpfulness | 0.10 | 0.76 |  |  |
|  | PSB-PD | 0.86 | 0.35 |  |  |
|  | PSB-PT | 1.74 | 0.19 |  |  |
|  | PSB-SR | 1.22 | 0.27 |  |  |
|  | PSB-SA | 0.04 | 0.85 |  |  |
| Self > Other X High > Low Loss Probability X Prosocial Tendency | LAS-Assessment | 0.00 | 0.95 |  |  |
|  | **LAS-Locomotion** | **15.73** | **0.00** | ******* | **Y** |
|  | **PSB-EC** | **14.74** | **0.00** | ******* | **Y** |
|  | PSB-Helpfulness | 0.73 | 0.39 |  |  |
|  | PSB-PD | 0.16 | 0.69 |  |  |
|  | PSB-PT | 3.95 | 0.05 |  |  |
|  | PSB-SR | 0.57 | 0.45 |  |  |
|  | PSB-SA | 1.55 | 0.21 |  |  |

***: p < 0.001, **: p < 0.01, *: p < 0.05.

LAS- Assessment: Locomotion Assessment Scale-Assessment; LAS- Locomotion: Locomotion Assessment Scale-Locomotion; PSB-EC: Penner Prosocial Battery-Empathic Concern

PSB- Helpfulness: Penner Prosocial Battery-Helpfulness; PSB- PD: Penner Prosocial Battery-Personal Distress; PSB- PT: Penner Prosocial Battery-Perspective Taking; PSB- SR: Penner Prosocial Battery-Social Responsibility; PSB- SA: Penner Prosocial Battery-Self-Reported Altruism.

**Table S3**

**ROI correlations per period with hoarding tendencies.**

| **Period &**  **Contrast** | **ROI** | **Difficulty Discarding** | **Emotional Attachment** | **Possessiveness** |
| --- | --- | --- | --- | --- |
| Viewing  Self > Other | L NAcc | 0.25 | 0.00 | -0.15 |
|  | R NAcc | 0.18 | -0.08 | -0.21 |
|  | R Ant. Insula | -0.17 | 0.01 | **-0.36*** |
| Work  Self > Other | L NAcc | -0.33^+^ | -0.13 | 0.06 |
|  | R NAcc | -0.29 | -0.11 | 0.08 |
|  | R Ant. Insula | -0.24 | -0.13 | 0.00 |
| Outcome  Self Trashed > All | L NAcc | **-0.49**** | **-0.43*** | -0.21 |
|  | R NAcc | **-0.41*** | **-0.36*** | -0.19 |
|  | R Ant. Insula | 0.00 | 0.02 | 0.01 |
| Outcome  Self Saved > Self Trashed | L NAcc | 0.359 | **.446*** | 0.083 |
|  | R NAcc | 0.277 | 0.347 | 0.015 |
|  | R Ant. Insula | 0.079 | 0.172 | 0.115 |

*Note.* Significant results are bolded. NAcc = nucleus accumbens; Ant. = Anterior; L = Left, R = Right. ***p* < .01, **p* < .05 two-tailed.


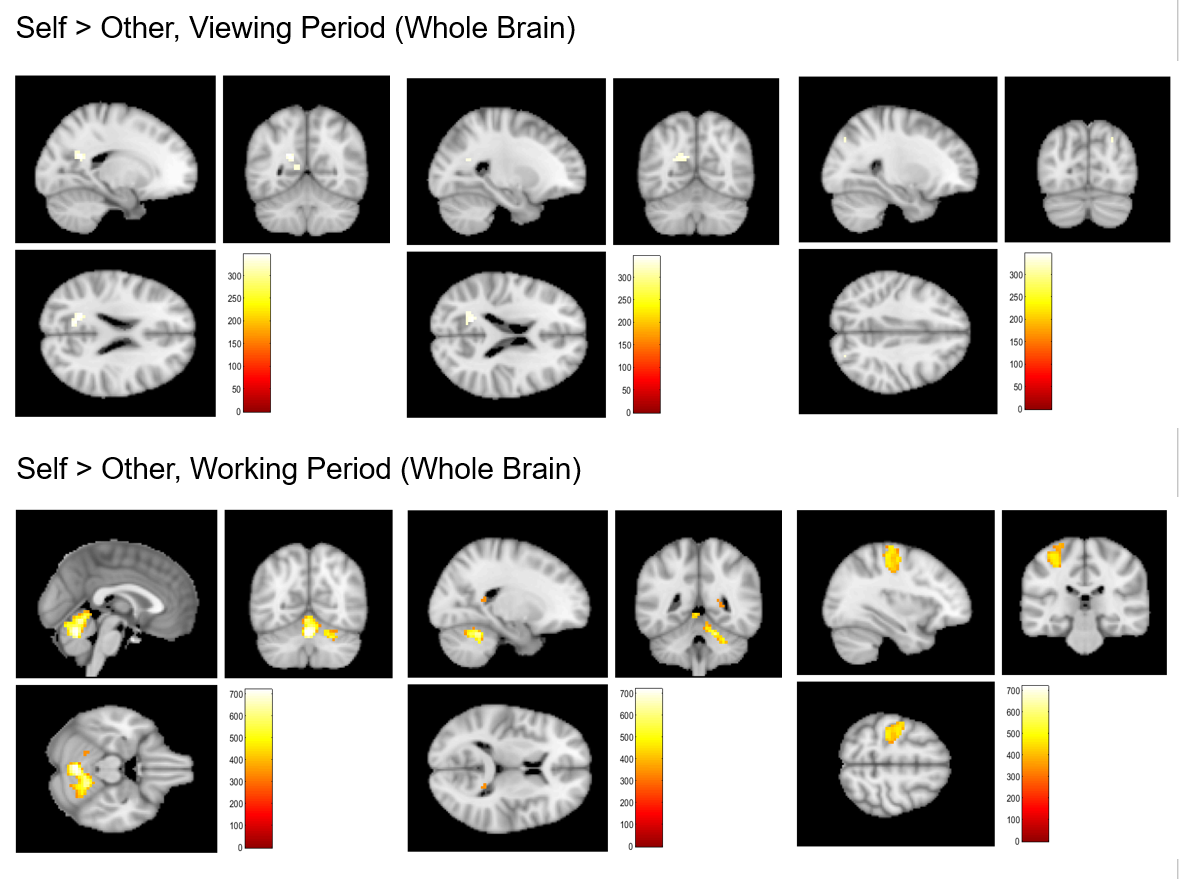


**Figure S1**

**Self > Other whole brain viewing and work period activation.**

*p* < .1 FWE TFCE, k > = 0.

**Table S4**

**Self > Other effects of work in the whole brain** **with the 2x2 model, masked by simple tapping**

| Period | Contrast | Region | Voxels | Peak equivZ | x | y | z |
| --- | --- | --- | --- | --- | --- | --- | --- |
| Work | Self > Other | Cerebellar Ant. Lobe/ Vermis | 221 | 3.35 | 0 | -61 | -22 |
|  |  |  |  | 3.24 | -3 | -52 | -7 |
|  |  |  |  | 2.95 | 12 | -55 | -16 |
|  |  | Cerebellar Ant. Lobe/ Cerebellar Cortex | 53 | 2.91 | 27 | -52 | -28 |
|  |  | Cerebellar Ant. Lobe/Cerebellar Cortex | 1 | 2.71 | 15 | -43 | -22 |
|  |  | RSC | 15 | 2.91 | 21 | -46 | 11 |
|  |  | RSC/ PHG/ Post. lateral ventricle horn |  | 2.47 | 30 | -55 | 8 |

Significant areas remaining from the Work period Self > Other contrast, masked by activation from the simple Tapping Localizer (tapping 2.0hz > rest, p < .001 uncorrected, k > 10), exclusive, at FWE < 0.1, k = 0. Ant. = Anterior; PHG = Parahippocampal Gyrus; Post. = Posterior; RSC = retrosplenial cortex.

**Table S5**

**Whole-brain analysis correlation with rated pretzel niceness**

| **Period** | **Regions** | **Voxels** | **Peak equivZ** | **x** | **y** | **z** |
| --- | --- | --- | --- | --- | --- | --- |
| Viewing | No voxels survive | | | | | |
| Work | Left Pre-CG | 11328 | 3.54 | -30 | -22 | 59 |
|  | Left Post-CG | - | 3.54 | -27 | -28 | 68 |
|  | Left Pre-CG | - | 3.54 | -45 | -13 | 53 |
|  | Right STG | 14 | 3.54 | 42 | 2 | -28 |
|  | Left MTG | 5 | 2.99 | -60 | -58 | 2 |
|  | Right extra-nuclear | 4 | 3.09 | 12 | 20 | -13 |
|  | Right MFG | 2 | 2.82 | 24 | 11 | 59 |
|  | Left Pre-CG | 14 | 3.24 | -57 | 2 | 32 |
|  | Right parahippocampal gyrus | 20 | 3.35 | 18 | -28 | -10 |
|  | Left MOG | 25 | 2.99 | -42 | -85 | 5 |
|  | Left MOG | - | 2.73 | -42 | -73 | 5 |
|  | Left MOG | - | 2.75 | -42 | -73 | 14 |
|  | Left IOG | 10 | 3.04 | -36 | -79 | -4 |
|  | Left IOG | 2 | 2.99 | -51 | -73 | 2 |
| Outcome | No voxels survive | | | | | |

*Note.* Threshold: *p* < .1 FWE TFCE, 0 voxels. Pre-CG = precentral gyrus, Post-CG = postcentral gyrus, STG = superior temporal gyrus, MTG = middle temporal gyrus, MFG = middle frontal gyrus, MOG = middle occipital gyrus, IOG = inferior occipital gyrus.

***
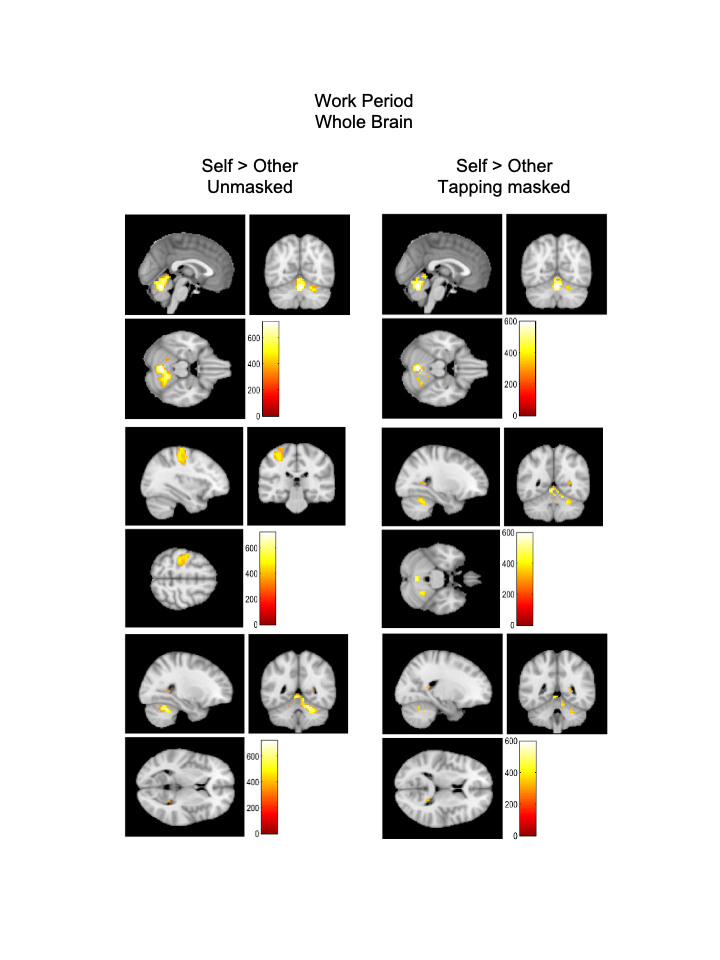
***

**Figure S2**

**Self > Other Work Activation Masked and Unmasked.** Whole brain analysis results from the work period comparing greater activation for self over other items before and after masking out significant areas of activation from the motor tapping localizer, to remove activation associated with tapping alone.

**
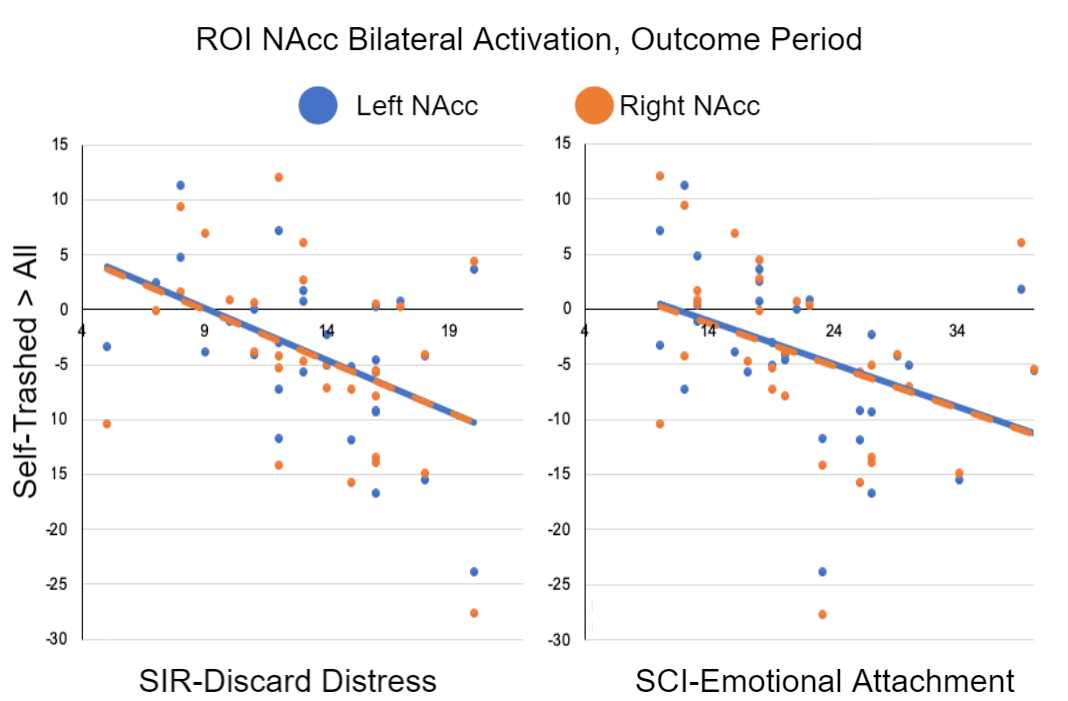
**

**Figure S3**

**ROI NAcc correlations with trait hoarding when items were saved.** When one’s own pretzels were trashed compared to all other outcomes, NAcc ROI activation significantly decreased with two hoarding tendencies: Saving Inventory Revised-discard distress and Saving and Cognitions Inventory-emotional attachment to possessions.

**Brain Imaging Task > Baseline Functional ROI**

Because there were some null results within our ROI and WB analyses, we added tests that looked for activation within the functional ROI mask of task > baseline in the whole-brain (Viewing and Work only).

**Viewing.** For a more refined test, we examined activation with a functional ROI of activation from task > baseline. For this Viewing period, masks were thresholded at *p* < .05 FWE, k > = 10, excluding occipital for greater precision. Differential activation associated with item creator (self > other) was then contrasted using the 2x2 model at *p* < .001 uncorrected, k > = 5. The functional mask of task > baseline during Viewing included precuneus, superior, middle, and inferior frontal gyrus (SFG, MFG, IFG), middle frontal gyrus, middle occipital gyrus, parahippocampal gyrus and paracentral lobule. Superior parietal lobule (SPL) was more active when viewing one’s own pretzels over others’, within the functional ROI of activation from task > baseline.

**Work.** During Work, tapping itself could increase activation in many regions. Thus, for greater precision, we used a more stringent functional threshold for Work, task > baseline, at *p* < .01 FWE, k > = 10, again excluding occipital. Task > baseline during Work included cerebellum, culmen, precentral gyrus (Pre-CG) and postcentral gyrus (Post-CG), and middle temporal gyrus (MTG). Within the work task > baseline functional ROI, one’s own pretzels increased activation compared to others’ in left insula, right culmen, and bilateral Pre-CG.

**Outcome.** We did not create a functional ROI of activation from task > baseline in the Outcome period.

**References**

1. Frost, R.O., Steketee, G., & Grisham, J. (2004). Measurement of compulsive hoarding: Saving Inventory-Revised. *Behavior Research and Therapy, 42*(10), 1163-1182.
2. Steketee, G., Frost, R., & Kyrios, M. (2003). Cognitive aspects of compulsive hoarding. *Cognitive Therapy and Research, 27*(4), 463-479.
3. Belk, R.W. (1985). Materialism: Trait aspects of living in the material world. *Journal of Consumer Research, 12*(3), 265-280.
4. Leonard-Barton, D. (1981). Voluntary simplicity lifestyles and energy conservation. *Journal of Consumer Research*, 243-252.
5. Penner, L.A. (2002). The causes of sustained volunteerism: An interactionist perspective. *Journal of Social Issues, 58*, 447-468.
6. Kruglanski, A.W., Thompson, E.P., Higgins, E.T., Atash, M.N., Pierro, A., Shah, J.Y., & Spiegel, S. (2000). To "do the right thing" or to "just do it": Locomotion and assessment as distinct self-regulatory imperatives. *Journal of Personality and Social Psychology, 79*, 793-815.
7. Tolin, D. F., Das, A., Hallion, L. S., Levy, H. C., Wootton, B. M., & Stevens, M. C. (2019). Quality of life in patients with hoarding disorder. *Journal of Obsessive-Compulsive and Related Disorders*, *21*, 55-59.
8. Hall, K. A. A., Stamatis, C. A., Shaw, A. M., & Timpano, K. R. (2019). Are hoarding symptoms associated with interpersonally relevant attentional biases? A preliminary investigation. *Journal of Obsessive-Compulsive and Related Disorders*, *22*, 100449.
9. David, J., Baldwin, P. A., & Grisham, J. R. (2019). To save or not to save: The use of cognitive bias modification in a high-hoarding sample. *Journal of Obsessive-Compulsive and Related Disorders*, *23*, 100457.
10. Cowles, D., & Crosby, L. A. (1986). Measure validation in consumer research: A confirmatory factor analysis of the voluntary simplicity lifestyle scale. *ACR North American Advances*.
11. Wertag, A., & Bratko, D. (2018). In search of the prosocial personality. *Journal of Individual Differences.* *40*(1), 55–62
12. Benjamin, L., & Flynn, F. J. (2006). Leadership style and regulatory mode: Value from fit?. *Organizational Behavior and Human Decision Processes*, *100*(2), 216-230.
